# Text Messaging Adherence Intervention for Adolescents and Young Adults with Chronic Kidney Disease: Pilot Randomized Controlled Trial and Stakeholder Interviews

**DOI:** 10.2196/19861

**Published:** 2020-08-14

**Authors:** Cyd Eaton, Margaret Comer, Cozumel Pruette, Kevin Psoter, Kristin Riekert

**Affiliations:** 1 Pulmonary & Critical Care Medicine Johns Hopkins University School of Medicine Baltimore, MD United States; 2 Division of Pediatric Nephrology Johns Hopkins University School of Medicine Baltimore, MD United States; 3 Division of General Pediatrics Johns Hopkins University School of Medicine Baltimore, MD United States

**Keywords:** medication adherence, mobile health, pediatrics, kidney diseases, kidney, mHealth, adherence, adolescent, young adult, intervention

## Abstract

**Background:**

Up to one-third of adolescents and young adults (11-21 years old) with chronic kidney disease exhibit suboptimal rates of adherence to renal-protective antihypertensive medications. Mobile health interventions may promote higher adherence to these medicines in these individuals, but empirical research is needed to inform best practices for applying these modalities.

**Objective:**

In this multiphase investigation, we developed and tested a theoretically informed text messaging intervention based on the COM-B model, a well-established health intervention framework stating that capability, opportunity, and motivation interactively modify health behaviors, to improve participants’ antihypertensive medication adherence in a pilot randomized controlled trial. Qualitative data on user experiences were obtained.

**Methods:**

In phase 1, intervention messages (Reminder+COM-B Message) were developed via stakeholder engagement of participants and pediatric nephrologists. In phase 2, the Reminder+COM-B Message intervention was tested against a Reminder-only Message active control condition in an 8-week pilot randomized controlled trial. The primary outcome was daily electronically monitored antihypertensive medication adherence and secondary outcomes included pre-post participant surveys of adherence self-efficacy, adherence barriers, outcome expectancies for taking medicine, and motivation for and importance of taking medicine. In phase 3, qualitative interviews related to user experiences were conducted with participants in the Reminder+COM-B Message intervention group.

**Results:**

Following phase 1, 34 participants (mean age 16.59 years, 41% female, 38% African American/Black, 35% hypertension diagnosis) completed the phase 2 pilot randomized controlled trial (n=18 in the Reminder+COM-B Message intervention group, n=16 in the Reminder-only Message active control group). All participants in the Reminder+COM-B Message intervention group completed a phase 3 qualitative interview. Overall, study procedures were feasible and the Reminder+COM-B Message intervention was acceptable to the participants (eg, 15/18 participants reported reading the majority of messages sent to them, 0/18 reported that the messages reduced their desire to take medicine). Prerandomization, there were no significant group differences in the rate of change in daily adherence over time. However, postrandomization, there was a significant group by time interaction (B=.01, *P*=.04) in which daily adherence decreased significantly over time in the Reminder-only Message active control group but remained stable in the Reminder+COM-B Message intervention group. There were no significant differences between groups in pre-post changes in survey responses. Qualitative interviews revealed participants’ perceptions of how the Reminder+COM-B Message intervention changed adherence behavior and highlighted several areas for improving the intervention (eg, adapt messaging timing, intensity, and content to match daily adherence, send praise when medicine is taken).

**Conclusions:**

The Reminder+COM-B Message intervention was feasible and acceptable to adolescents/young adults and demonstrated potential to promote participants’ daily medication adherence beyond simple reminders. Further research is needed to determine the Reminder+COM-B Message intervention’s mechanisms of adherence behavior change and to incorporate qualitative participant feedback into a modified version of this intervention to enhance its acceptability.

**Trial Registration:**

ClinicalTrials.gov NCT03651596; https://clinicaltrials.gov/ct2/show/NCT03651596

## Introduction

Several antihypertensive medications serve as renal protective agents by lowering blood pressure or decreasing urinary protein excretion with the goal of slowing disease progression in children with chronic kidney disease (CKD) [1,2]. However, up to one-third of adolescents and young adults (AYAs) with CKD do not consistently take these medicines [3]. AYAs with CKD could benefit from behavior intervention to improve antihypertensive medication adherence, which in turn could improve long-term health outcomes. Adherence interventions exist for AYAs with other chronic medical conditions [4], but typically involve face-to-face visits, thereby limiting efficient dissemination and patient access [5]. Mobile health (mHealth) interventions offer solutions to these barriers by delivering the intervention to nearly anyone with a smartphone, including 95% of adolescents in the United States [6]. However, to date, there has been very little rigorous empirical investigation of mHealth adherence intervention efficacy or the application of mHealth approaches for AYAs with CKD [7,8].

mHealth interventions have been developed for medication adherence in children and AYAs with other medical conditions (eg, sickle cell disease, asthma, type 1 diabetes, migraine). However, these interventions have primarily relied on daily dose reminders [9-11], which show short-term effects on adherence and have barriers to long-term practicality (eg, repeated reminders could be viewed as intrusive instead of helpful) [12,13], or involve self-tracking daily adherence (eg, self-reported logs or video recordings of taking medicine), which have low engagement and completion rates [14,15]. Other limitations to existing interventions include no evidence for their efficacy on objective adherence behavior [10,16], no theoretical basis to an a priori outline of the intervention’s mechanisms of behavior change [17], relying on components with barriers to long-term sustainability (eg, monetary incentives for taking medicine) [15], or not actively involving stakeholders in intervention development and refinement, which is essential for AYA engagement and intervention uptake [18].

The aim of the current study was to address these limitations of prior interventions by developing and testing a theoretically informed antihypertensive medication adherence-promoting mHealth messaging intervention for AYAs with CKD using objective adherence outcomes and qualitative stakeholder feedback. Our messaging intervention was based on the COM-B model, a well-established health intervention framework stating that *capability* (ie, skills, knowledge, and ability to complete a behavior), *opportunity* (ie, environmental factors prompting a behavior), and *motivation* (ie, conscious, reflective, and learned reasons for engaging in a behavior) interactively modify health *behaviors*, including adherence [19]. Opportunity is often targeted in mHealth interventions via medicine reminder messages. Longitudinal research involving AYAs with CKD has shown that higher capability and motivation to adhere to medications is associated with higher objective antihypertensive medication adherence [20]. Hence, it was expected that sending a reminder message (adherence opportunity) with additional messaging aimed at improving AYAs’ adherence capability (eg, teach skills to reduce barriers and improve adherence self-efficacy) and motivation (eg, highlight the benefits of adherence to activate desire to take medicine) would improve medication adherence, which formed the basis for the design of our Reminder+COM-B Message intervention.

In the current study, we conducted a mixed methods investigation, which involved (1) developing the Reminder+COM-B Message intervention for antihypertensive medication adherence in AYAs with CKD (Phase 1), (2) preliminarily evaluation of the Reminder+COM-B Message intervention against a Reminder-only Message active control condition in an 8-week pilot randomized controlled trial (RCT; Phase 2), and (3) obtaining poststudy qualitative feedback from AYAs randomized to the Reminder+COM-B Message intervention (Phase 3). We hypothesized that (1) study procedures would be feasible (few to no technical issues implementing study procedures, low cost), and acceptable and engaging to the majority of AYAs (ie, few to no AYA-reported issues with study procedures in either group, majority of AYAs in the Reminder+COM-B Message intervention group would report reading or noticing the messages sent, <10% of AYAs would report that the Reminder+COM-B Message intervention reduced their desire to take medicine); (2) postrandomization (Phase 2), daily adherence (dose taken or not) would show a faster rate of improvement in the Reminder+COM-B Message intervention group compared to the Reminder-only Message active control group; and (3) pre-post survey scores representing AYA perceptions of adherence motivation and capability would show greater improvement in the Reminder+COM-B Message intervention group compared to the Reminder-only Message active control group (Phase 2). Given conventions for analyzing adherence as an overall mean, we also examined overall mean changes in adherence from baseline to postrandomization according to group allocation (Phase 2). During qualitative interviews (Phase 3), we probed AYAs’ perceptions on the Reminder+COM-B Message intervention’s mechanisms of behavior change and suggestions for improving its acceptability.

## Methods

### Procedure

#### Recruitment

The Johns Hopkins School of Medicine Institutional Review Board approved all study procedures prior to recruitment, which occurred at a single pediatric nephrology clinic from October 2018 to November 2019. Trained study staff identified potentially eligible AYAs via clinic roster and electronic medical record review. Inclusion criteria were: aged 11-21 years old, CKD diagnosis, current antihypertensive medication prescription (pill form only), and access to a mobile phone that received text messages. Exclusion criteria were: underwent solid organ transplantation, received dialysis, had a sibling participating in the study, unable to understand spoken English, had a developmental delay or significant cognitive impairment precluding their ability to complete study procedures, or declined to use the electronic adherence monitoring device or to be audio-recorded during qualitative interviews. Potentially eligible AYAs were mailed or emailed a letter describing the study and providing the opportunity to opt out of recruitment. AYAs who did not opt out were contacted by telephone to provide study details, conduct further eligibility screening, and, if eligible and interested in enrolling, coordinate informed consent/assent procedures. Informed consent/assent procedures were conducted with participants during a telephone call with study staff (AYAs >18 years old provided consent for themselves; for AYAs <18 years old, a primary caregiver provided informed consent for their child and the AYA provided informed assent for themselves).

#### Phase 1: Reminder+COM-B Message Intervention Development

Message content was developed by the study team (experts on AYA medication adherence, behavior change theory, and pediatric nephrology) to target COM-B model components [19] and incorporate effective public health communication strategies; specifically, framing messages based on what the AYA could gain by taking their medicine as opposed to what they could lose by not taking their medicine, minimizing use of fear appeals, and incorporating novelty and relevance to the intended audience [21-24] (eg, providing different content each day, gearing content toward AYAs with CKD taking antihypertensive medicine, writing content at reading level and style appropriate for age group). Poor framing of health messages can have iatrogenic effects by reducing engagement in the targeted behavior [25]. Messages were written at the ≤5th-grade reading level and contained <140 characters. Messages targeting the COM-B model’s capability and motivation components were based on validated self-report measures of AYA adherence barriers and beliefs [26,27]. The COM-B model’s opportunity component was targeted with a simple medicine reminder message. Pediatric nephrologists (N=6) at the study site provided feedback on the messages’ medical accuracy.

The initial intervention message pool was presented to 10 AYAs with CKD (mean age 16.50, SD 3.41, range 12-21 years) during a semistructured telephone interview (~60 minutes; audio-recorded). AYAs rated messages on acceptability, effectiveness, helpfulness, and comprehension, and provided open-ended suggestions for improving content. AYAs received US $20 each for completing the interviews.

Messages rated by <85% of AYAs as acceptable, effective, helpful, or understood were excluded or revised before inclusion in the final pool tested in Phase 2. Messages were revised based on interview responses and suggestions, including adjusting the message wording for enhanced comprehension and age appropriateness, and adding new content generated by AYAs. The final Reminder+COM-B Message pool included 14 messages targeting adherence capability (eg, “Tip: Put your medicine in a safe place you look each day [like the kitchen counter or by your bed] to help remember to take them”), 14 messages targeting adherence motivation (eg, “Think about your future goals and how being healthier by taking your medicine may help you achieve them!”), and a simple reminder targeting adherence opportunity (“Please remember to take your medicine”). The Reminder+COM-B Message intervention involved sending AYAs a daily message bundle, which included the opportunity message (simple reminder) and a capability or motivation message (alternated each day) at the time(s) when the AYA reportedly took their antihypertensive medicine. All participants in this group received the same Reminder+COM-B Message bundle in the same order (eg, opportunity+capability message bundle on day 1, opportunity+motivation message bundle on day 2). If the participant was prescribed a twice-a-day antihypertensive medication regimen, they received the Reminder+COM-B Message bundle with their first dose and the Reminder-only Message with their second dose.

##### Reminder-Only Message Active Control Condition

The Reminder-only Message active control condition involved sending AYAs the daily opportunity message only (the same simple reminder). Reminder messages were sent at the time(s) when each AYA reportedly took their antihypertensive medicine. If the participant’s antihypertensive medication regimen was twice a day, they received a separate reminder message when each expected dose was due.

#### Phase 2: Pilot RCT

AYAs were mailed electronic pill bottles for monitoring adherence (see Objective Medication Adherence section). After the bottle was delivered, study staff conducted a telephone call with the AYA to describe how the bottle worked, transfer the monitored antihypertensive medicine into the bottle, and answer AYA or caregiver questions. AYAs were instructed to use the study bottle for their antihypertensive medicine during the 8-week pilot RCT and to transfer any refills to the study bottle during this period. AYAs were informed that the study text messages would be sent to their mobile phone at some point during the pilot RCT but were not provided with a specific date. Text messages (all one-way) containing the Reminder+COM-B Message intervention or Reminder-only Message active control content were sent from a single study number via REDCap’s Twilio interface (research team costs included US $1/month for the phone number and US $0.007/outgoing text message; participants used their personal cellular devices and plans). A brief demographic survey was sent to AYAs ≥18 years old and caregivers of AYAs <18 years old via REDCap. AYAs completed online surveys (Qualtrics) at baseline and after the pilot RCT ended.

The first 4 weeks of the pilot RCT (baseline) evaluated AYA adherence without sending any messages. AYAs were randomized to the Reminder+COM-B Message intervention or Reminder-only Message active control group and received their respective group’s messages for the last 4 weeks of the pilot RCT. AYAs were randomized at a 1:1 basis to either group at the end of baseline. A random number generator was used for simple randomization and AYAs were assigned consecutively to a group. Blinding the study team to group assignment was not possible; however, the messages were delivered remotely via text message rather than face to face. At the end of the 8 weeks, AYAs in the Reminder+COM-B Message intervention group completed a qualitative interview (see Qualitative Interviews section). AYAs in both groups received US $40 each for completing the pilot RCT and pre and postsurveys.

##### Objective Medication Adherence

AdhereTech bottles electronically assessed daily antihypertensive medication adherence via a cellular-connected cap that recorded the date and time when the bottle cap was opened and closed. Timestamps were automatically transferred to AdhereTech’s secure online portal. If more than one antihypertensive medication was prescribed, AYAs selected which medicine they wanted to monitor in the AdhereTech bottle. At the end of the monitoring period, the study team debriefed with participants to query for major problems when using the AdhereTech bottle (eg, issues that led them to stop using the device) and whether the monitored medicines were taken from a container other than the AdhereTech bottle during the study period [28]. Daily adherence was coded as whether the bottle was opened that day (1) or not (0). If the AYA took the monitored medicine twice a day, daily adherence was coded as whether the bottle was opened twice that day (1) or not (0). Overall mean adherence was calculated as the number of bottle cap openings recorded divided by the total number of expected openings based on the prescribed regimen and days in the monitoring period.

#### Surveys of Adherence Capability and Motivation

##### Adherence Capability

To represent capability as defined in the COM-B model (eg, skills, knowledge, ability to take medicine) [19], the Riekert Self-Efficacy Scale [20] and the Adolescent Medication Barriers Scale (AMBS) [26] were administered.

The Riekert Self-Efficacy Scale (12 items) asked AYAs to rate their ability to take medicine in different situations on a 10-point Likert scale ranging from “not at all sure” to “completely sure” (eg, “How sure are you that you can take your blood pressure medicine the way your doctor said when you want to do something else?”). Ratings were summed and divided by 12 for a scaled score ranging from 1 to 10 (higher scores indicate higher self-efficacy). Internal consistency (Cronbach α) in this study ranged from .94 to .95.

The AMBS (17 items) assessed AYAs’ adherence barriers. AYAs rated each item using a 5-point Likert scale ranging from “strongly disagree” to “strongly agree” (eg, “I find it hard to stick to a fixed medication schedule”). The AMBS contains subscales but only the total score was analyzed in this study (ratings were summed and divided by 17 for a scaled score ranging from 1 to 5; higher scores indicate higher adherence barriers). Internal consistency in this study ranged from .81 to .89.

##### Adherence Motivation

To represent motivation as defined in the COM-B model (conscious, reflective, and learned reasons for taking medicine as prescribed) [19], several scales from the Beliefs About Medication Scale [27,29,30] were administered.

The Positive Outcome Expectancies (POE; 20 items) and Negative Outcome Expectancies (NOE; 13 items) scales assessed AYAs’ expectations of favorable/unfavorable outcomes for taking medications as prescribed (eg, “When I take my medicine the way I should, I feel well enough to do things I enjoy” [POE]; “Taking my medicine the way I should makes me miss out on doing fun things” [NOE]). Items are rated on a 7-point Likert scale ranging from “definitely do not agree” to “definitely agree.” Item ratings were summed and divided by the number of scale items to obtain scaled scores ranging from 1 to 7 (higher scores indicate more positive expectations [POE] or more negative expectations [NOE]). Internal consistency in this study ranged from .85 to .87 for POE and from .90 to .92 for NOE.

The Adherence Motivation and Importance scales (3 items each) assessed AYAs’ perspectives on the importance of taking medicine (eg, “How important do you think it is for you to take your blood pressure medication the way the doctor said when you feel just fine?”) and motivation to do so (eg, “How much do you want to take your blood pressure medication the way the doctor said everyday?”) on a 10-point Likert scale. Item ratings were summed and divided by 3 to obtain scaled scores (higher scores indicate higher importance or motivation). Internal consistency in this study ranged from .76 to .96 for Importance and from .84 to .96 for Motivation.

#### Phase 3: Qualitative Interviews

Following Phase 2 completion, AYAs in the Reminder+COM-B Message intervention group were invited to complete audio-recorded and transcribed qualitative interviews (~30 minutes) by telephone. The interviewer (CE) followed an iterative interview guide evaluating AYAs’ perceptions of the intervention, including perspectives on message acceptability and engagement (eg, “Did you read all of the messages?” “In what, if any, ways did the messages make you not want to take your medicine?”), mechanisms of behavior change, and suggestions for improving the intervention. Interviews lasted, on average, 31 minutes (SD 11). No repeat interviews were conducted. AYAs received US $20 for their time.

### Data Analysis

The primary feasibility, acceptability, and engagement results are reported as proportions and percentages. Descriptive statistics (mean, SD, range) were calculated for primary study variables by randomization group and measurement time point. For hypothesis testing, statistical significance was assumed when *P*<.05. A linear mixed model (PROC MIXED; SAS 9.4 Software, Cary, NC, USA) was used to examine whether changes in daily adherence over time differed by group allocation, controlling for AYA age, gender, race, and hypertension diagnosis (covariates were based on variables commonly associated with pediatric adherence [4], including AYAs with CKD [20]). Change in daily adherence during both the baseline and postrandomization phases were initially examined to determine the functional form of the time variable (linear or quadratic) and whether to include an individual-level random slope. The best-fitting model for time was selected using the Akaike information criterion (AIC) values [31]. Based on this initial examination, time was modeled as a linear function with an individual-level random slope during baseline (AIC=750.6) and postrandomization (AIC=750.9). When time was modeled as a quadratic function with an individual-level random slope, the AIC value was 765.8 during baseline and during postrandomization. Models were fitted using restricted maximum likelihood estimation. An autoregressive covariance structure was used to account for expected correlations between repeated daily adherence assessments within participants. There were no investigator-identified or participant-reported technical problems when transmitting data. In instances when a participant reported taking their antihypertensive medicine from a different container than the electronic pill bottle with specific dates (n=3), these data were imputed; thus, missed doses were assumed to be medication nonadherence and not missing data.

Repeated-measures analysis of variance (IBM SPSS Statistics Version 26) with one within-subject factor (time point) and one between-subject factor (treatment group) was used to examine whether the change in overall mean adherence during baseline and postrandomization and pre-post mean survey scores differed by group (controlling for AYA age, gender, race, and hypertension diagnosis [4,20]). The magnitude of within-treatment group change between study time points was examined with Cohen *d*.

All qualitative interview transcripts were content-analyzed to identify major themes. The codebook was developed by the principal investigator and study team. Each interview was coded separately by two coders (CE and MC) in nVIVO Pro v.11 (QSR International). The coders identified, discussed, and resolved coding discrepancies in each transcript. The study authors reviewed final codes to identify the number of AYAs reporting that they read the messages, noticed the messages were sent and delivered (including AYAs who reportedly did not read the message content), and perceived the Reminder+COM-B Message intervention to reduce their desire to take their medicine, as well as AYA perceptions of the intervention’s mechanisms of behavior change and suggestions for improving it.

## Results

### Participants

**Figure 1:**
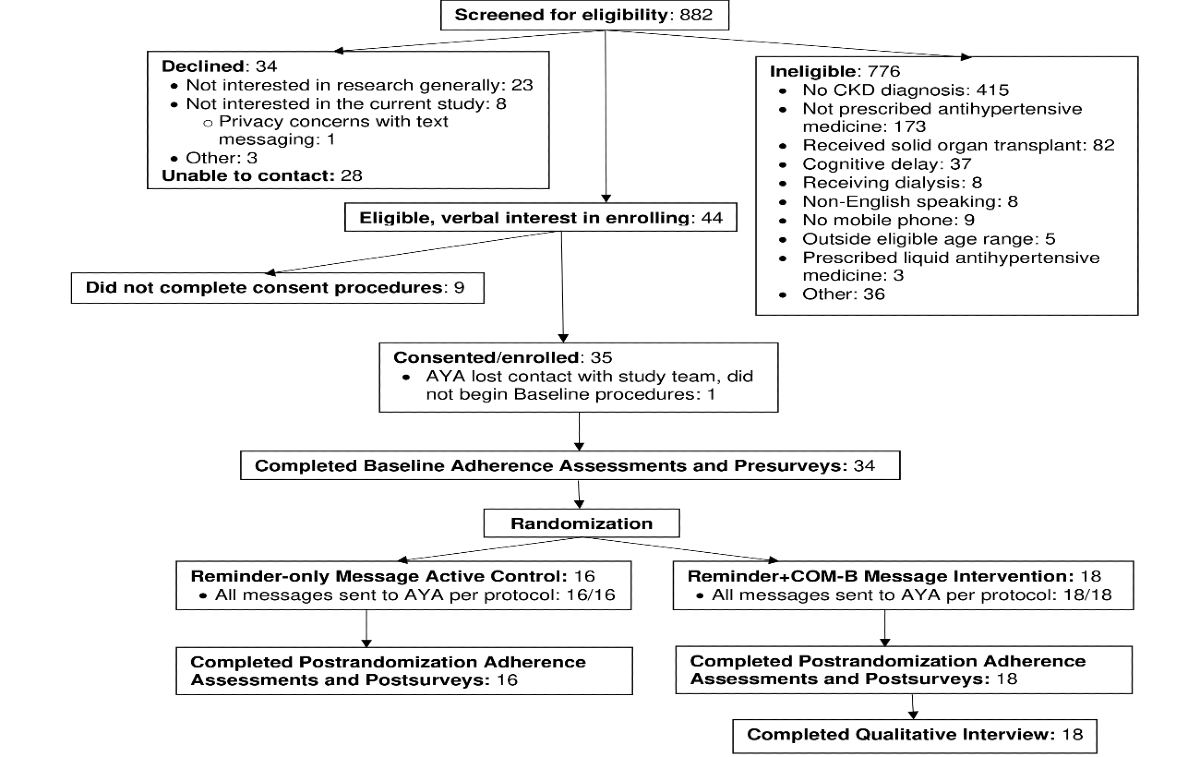
Consolidated Standards of Reporting Trials (CONSORT) flow diagram of screening, recruitment, and enrollment of potentially eligible participants and study procedure completion for enrolled adolescents and young adults (AYA). CKD: chronic kidney disease.

Figure 1 shows the Consolidated Standards of Reporting Trials (CONSORT) diagram for screening, enrollment, and overall study participation. Of the potentially eligible AYAs, 34 declined to enroll. Of the 44 AYAs who were screened as eligible and initially expressed verbal interest in enrolling, 35 AYAs completed informed consent/assent procedures to enroll. There were no significant differences in age, gender, or race between those who enrolled and those who declined to enroll.

Of the 35 enrolled AYAs, one lost contact with the study team after enrolling and did not complete any further procedures. Hence, 34 AYAs (mean age 16.59, SD 3.26, range 11-21 years) were randomized in Phase 2 (Reminder+COM-B Message intervention group n=18, Reminder-only Message active control group n=16). The final sample was primarily male, White, without a hypertension diagnosis, and taking an angiotensin-converting enzyme inhibitor once a day (see Table 1 for detailed demographic characteristics). There were no statistically significant differences in demographic characteristics by randomization group allocation.

**Table 1:** Demographic characteristics of participants.

Variable		Overall Sample (N=34)	Reminder-only Message Active Control (n=16)	Reminder+COM-B Message Intervention (n=18)
Age (years), mean (SD)		16.59 (3.26) (range 11-21)	16.13 (3.44)	17.00 (3.22)
**Gender n (%)**				
	Female	14 (41)	7 (44)	7 (39)
	Male	20 (59)	9 (56)	11 (61)
**Race/ethnicity, n (%)**				
	White	19 (56)	8 (50)	11 (61)
	African American/Black	13 (38)	7 (44)	6 (33)
	Other	2 (6)	1 (6)	1 (6)
**Hypertension diagnosis, n (%)**				
	Yes	12 (35)	5 (31)	7 (39)
	No	22 (65)	11 (69)	11 (61)
**Hypertension medication, n (%)**				
	ACE^a^ inhibitor	28 (82)	14 (87)	14 (78)
	Other^b^	6 (18)	2 (13)	4 (22)
**Dose frequency, n (%)**				
	Once a day	32 (94)	15 (94)	17 (94)
	Twice a day	2 (6)	1 (6)	1 (6)

^a^ACE: angiotensin-converting enzyme.

^b^“Other” hypertension medicines included calcium channel blocker or angiotensin II receptor blocker.

### Feasibility, Acceptability, and Engagement

Only 9 AYAs were ineligible to be enrolled in the study due to not having a mobile phone. No AYAs were ineligible due to having a mobile phone that did not receive text messages. No AYA declined to participate due to concerns about the expense of receiving study text messages on their mobile phone. All AYAs in the final sample (34/34, 100%) completed the Phase 2 pilot RCT procedures and all AYAs in the Reminder+COM-B Message intervention group (18/18, 100%) completed a Phase 3 qualitative interview. The decline rate for study enrollment was relatively high (34/106, 32.1%), although the primary reason for declining was lack of interest in research participation generally (23/34, 68%). For AYAs who declined for reasons related to this study, the primary reason was discomfort discussing topics related to having a medical condition during qualitative interviews (n=3); one person cited concerns with privacy related to text messaging and one person reported that they did not want their adherence to be monitored (the concern was specific to adherence monitoring, not the monitoring device itself). However, retention was high for AYAs who enrolled and began the pilot RCT in Phase 2 (34/34, 100%).

All AYAs (34/34, 100%) reportedly used the electronic pill bottles without indicating major problems that led them to stop using the device. The research team did not identify any technical issues with data transmission from the electronic pill bottles to the data collection portal. The text messaging plan cost the research team up to US $0.59 per AYA for 1 month plus US $1.00/month for the dedicated phone number used to send text messages to all participants.

No AYAs in either group (0/34) asked for the messages to stop or reported that the messages bothered them. The majority of AYAs in the Reminder+COM-B Message intervention group reportedly read the messages sent to them (15/18, 83%) and all AYAs in this group (18/18, 100%) reportedly noticed that the messages were sent and received (even if they did not read the content). No AYAs in the Reminder+COM-B Message intervention group (0/18) reported that the messages reduced their desire to take their medicine.

### Changes in Daily Adherence by Group Over Time During the Phase 2 Pilot RCT

Detailed linear mixed model results are shown in Table 2. In the final models accounting for covariates, daily adherence did not significantly change over time during baseline and there was no significant group by time interaction. As no significant group by time interaction was observed for adherence slopes during baseline, the decision was made to report changes in daily adherence separately during baseline and postrandomization.

**Figure 2:**
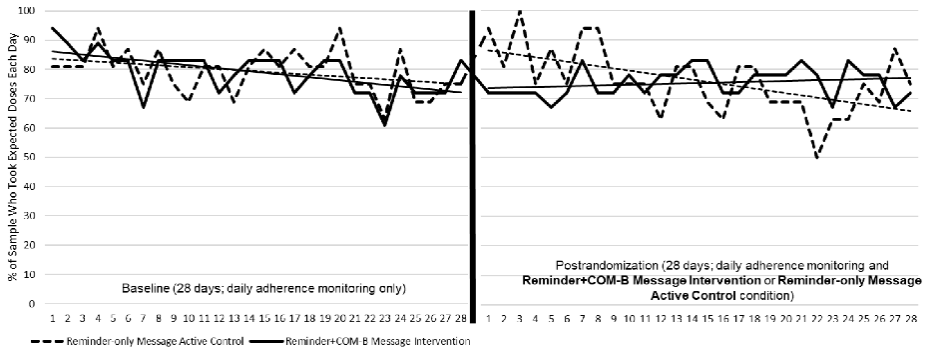
Daily objective medication adherence by randomization group during the baseline and postrandomization phases. The y-axis refers to the percentage of the sample who were recorded as having taken their medicine dose(s) each day.

Postrandomization, daily adherence significantly decreased over time in the overall sample but there was a significant group by time interaction. Specifically, the Reminder-only Message active control group showed a higher initial response to receiving the text messages and then a steeper decline in daily adherence over the postrandomization phase compared to the Reminder+COM-B Message intervention group’s daily adherence, which remained relatively stable with some improvement over time (see Figure 2).

**Table 2:** Changes in adolescent/young adults’ daily objective medication adherence by randomization group over time.

Effect			Baseline phase (4 weeks)						Postrandomization phase (4 weeks)				
			Estimate^a^ (SE)		95% CI/*z*^b^		*P* value		Estimate (SE)		95% CI/*z*^b^		*P* value
**Fixed effects**													
	Intercept	.91 (.08)		.75- 1.06		<.001		.95 (.10)		.75-1.15		<.001	
	Time (Day)	–.003 (.003)		-.01-.003		.27		–.01 (.003)		–.01-.001		.02	
	Group^c^	.03 (.08)		–.12-.18		.71		–.13 (.10)		–.33-.06		.18	
	Age	.002 (.01)		–.02-.02		.86		–.01 (.01)		–.04-.02		.47	
	Gender^d^	–.07 (.07)		–.21-.07		.35		.005 (.09)		–.18-.19		.96	
	Hypertension diagnosis^e^	–.10 (.07)		–.25-.05		.19		–.08 (.10)		–.27-.11		.42	
	Race^f^	–.003 (.07)		–.14-.14		.97		–.11 (.09)		–.30-.07		.23	
	Group × Time	–.002 (.004)		–.01-.01		.65		.01 (.004)		.0004-.02		.04	
**Random effects**													
	Intercept	.03 (.01)		2.60		.005		.06 (.02)		3.05		.001	
	Random slope	<.001 (<.001)		2.29		.01		<.001 (<.001)		2.26		.01	

^a^Estimate is beta for fixed-effects models and is variance for random-effects models.

^b^95% CI for fixed-effects variables and *z* for random-effects variables.

^c^Randomization group was coded as Reminder-only Message active control=0, Reminder+COM-B Message intervention=1.

^d^Gender was coded as male=0, female=1.

^e^Hypertension diagnosis was coded as no hypertension=0, hypertension=1.

^f^Race was coded as White=0, non-White=1.

#### Posthoc Sensitivity Analysis for Baseline Adherence

A posthoc sensitivity analysis was conducted to exclude the first 7 days of adherence data due to potential Hawthorne effects of knowing one’s adherence was being electronically monitored. Results were similar whether the first 7 days of baseline data were excluded or included (adherence demonstrated a nonsignificant decline over time, with no significant group by time interaction).

### Changes in Overall Mean Adherence and AYA Pre-Post Surveys During the Pilot RCT

During baseline, overall mean adherence was 80.60% (SD 22.47) in the Reminder-only Message active control group and was 79.46% (SD 22.62) in the Reminder+COM-B Message intervention group. During postrandomization, overall mean adherence was 76.76% (SD 27.31) in the Reminder-only Message active control group and 76.00% (SD 27.00) in the Reminder+COM-B Message intervention group. There were no significant differences between treatment groups in changes in overall mean adherence by measurement time point (no group by time interactions) or in mean survey scores. The magnitudes of within-group mean changes between time points were small (all Cohen *d*<.25). Table 3 shows the mean scores for survey responses by group allocation and measurement time point.

#### Posthoc Sensitivity Analysis for Mean Baseline Adherence

A posthoc sensitivity analysis was conducted to exclude the first 7 days of adherence data from the baseline mean scores due to potential Hawthorne effects of knowing that one’s adherence was being electronically monitored. Results were similar whether the first 7 days of baseline adherence data were excluded or included (no significant group by time interactions).

**Table 3:** Descriptive data for mean medication adherence and adolescent/young adult survey scores by group allocation and time point.

Variable			Baseline				Postrandomization			*P* value^a^		Cohen *d*^b^	
			mean (SD)		Range		mean (SD)		Range				
**Overall medication adherence**											.97		
	Active control^c^	80.60 (22.47)		32.14-100		76.76 (27.31)		21.43-100				.15	
	Intervention^d^	79.46 (22.62)		35.71-100		76.00 (27.00)		7.14-100				.14	
**Medication self-efficacy**											.57		
	Active control	7.90 (1.74)		4.08-9.75		8.03 (1.88)		3.83- 9.83				–.07	
	Intervention	7.95 (1.95)		3.33- 9.67		7.83 (2.36)		2.92- 10.00				.06	
**Positive outcome expectancies**											.42		
	Active control	5.81 (0.56)		5.05- 6.75		5.85 (0.40)		5.30- 6.80				–.08	
	Intervention	5.70 (1.00)		4.00-7.00		5.94 (0.90)		3.85-7.00				–.25	
**Negative outcome expectancies**											.61		
	Active control	2.07 (1.12)		1.00-4.31		2.19 (1.24)		1.00-5.31				–.10	
	Intervention	1.72 (0.94)		1.00-4.62		1.91 (1.09)		1.00-5.46				–.19	
**Barriers to adherence**											.79		
	Active control	2.23 (0.55)		1.35-3.41		2.25 (0.69)		1.12-3.65				–.03	
	Intervention	1.94 (0.56)		1.18-2.94		1.92 (0.66)		1.00-3.35				.03	
**Motivation for adherence**											.46		
	Active control	7.88 (2.41)		2.00-10.00		8.44 (2.92)		1.00-10.00				–.21	
	Intervention	8.30 (2.44)		1.00-10.00		7.87 (2.71)		3.33-10.00				.17	
**Importance of adherence**											.18		
	Active control	9.25 (1.72)		3.33-10.00		8.81 (1.85)		3.33-10.00				.25	
	Intervention	9.24 (1.58)		4.00-10.00		9.19 (1.98)		3.33-10.00				.03	

^a^*P* values are for group by time interactions for changes in mean adherence or surveys.

^b^Cohen *d* reflects the magnitude of change within randomization group from baseline to postrandomization.

^c^Reminder-only Message active control group.

^d^Reminder+COM-B Message intervention group.

### Qualitative Interviews with AYAs in the Reminder+COM-B Message Intervention Group

#### Mechanisms of Adherence Behavior Change

The majority of AYAs tended to view all Reminder+COM-B Message bundles irrespective of content as cues to take medicine: *“*When I got the text, I would look at my phone and then remembered that it was time to take the medicine” (14-year-old male). However, a 17-year-old male pointed out that the different content helped him attend to the messages: *“*Because [the messages] are different every time, I thought, ‘I’m going to read this and then I’m going to take my medicine.’ So it did help that they were different.”

Occasionally, AYAs recalled messages that provided helpful content regarding capability (skills) or motivation to maintain adherence. For example, an 18-year-old female discussed how the messages enhanced her adherence motivation: “I think [the messages] helped me by reading them and then thinking over that it is really important for me to take [my medicine] and it does motivate me to take it.” Other key quotes are shown in Textbox 1.

**Additional qualitative adolescent/young adults’ feedback about the Reminder+COM-B Message intervention.**

**Theme 1:** **Mechanisms of behavior change**

- Subtheme: Reminder+COM-B Message intervention bundles are cues to take medicine irrespective of content

When the messages were sent, it was a reminder to take my medicine. I looked at it and said, “I’ve got to go take my medicine.”

16-year-old male

[The messages] gave me a reminder, like, “Oh, I need to take my medicine.” And I would usually do it after I got the text message.

19-year-old female

[The messages are] sort of like a reminder on your phone.

15-year-old male

- Subtheme: Message content facilitated behavior changes to support adherence

There was one [message] about planning ahead for me not being at home. That helped me a lot...because on the weekends and Fridays, I’m away from home. I made sure I set another reminder for the time when I knew I’d get home. That way, I’d get a reminder fairly quick that I should take my medicine.

14-year-old male

When I got that message [about refilling the prescription], it actually made me do a double take and look and see how much medicine I had and if I needed to get a refill or not.

18-year-old male

**Theme 2:** **Suggestions to improve intervention acceptability**

- Subthemes: Adjust Reminder+COM-B Message intensity, timing, and content to match adherence behavior, barriers, and adolescent/young adult availability, receptivity, and engagement

Gauge how frequent to send the messages based on how consistent the person is with taking the medicine.

18-year-old male

I think that you should send [the messages] twice because sometimes people don’t get them.

14-year-old female

At first, I thought, “These are useful.“ And then because it happened every day, I started getting a little bit annoyed - but [the messages] did help me on the weekends [when] I forget.

17-year-old male

I forget to check my phone some of the time. When we would go to a friend’s house, I wouldn’t get [my phone] or bring it. And when we got home, I would be tired. Maybe we could set [the messages] at a different time or set more than one reminder to [take my medicine].

11-year-old female

[Send messages] at a time when the person is up and able to look at their phone.

18-year-old female

#### AYA Suggestions for Improving the Reminder+COM-B Message Intervention Acceptability

Tailoring message intensity, timing, and content to correspond to actual adherence behavior, current adherence barriers, or AYA availability, receptivity, and engagement could improve the intervention’s acceptability. A 21-year-old male suggested,

If you see a trend of a person taking their medicine spot-on, lessen the amount of messages. If you see that they're not taking the medicine, make the reminders more repetitive and more prominent.

Sending too many messages could lead to disengagement: “I wasn’t really paying attention. I get like 1000 messages a day” (11-year-old male). Providing positive reinforcement for taking medications as prescribed could also improve intervention acceptability: “I think if they did take it, they should [get a message saying], ‘Good job taking your medicine and don't forget next time.’ Because I think people will feel good inside” (14-year-old female). Message content should ideally match the AYA’s current situation and potential barriers to adherence: “Maybe if you’re running low, that message [about getting a refill] pops up, but if your pill bottle is completely full, that message doesn’t really help you” (21-year-old male). Other key quotes appear in Textbox 1.

## Discussion

### Principal Findings

The current study preliminarily investigated a new mHealth messaging intervention based on the COM-B model [19] that aimed to promote objectively measured antihypertensive medication adherence in AYAs with CKD and obtained qualitative stakeholder feedback on user experiences. The Reminder+COM-B Message intervention was feasible, acceptable, and subjectively engaging to participants. Our pilot RCT results suggest that this intervention had stabilizing effects on daily adherence compared to a Reminder-only Message active control group. Qualitative interviews provided insight into the Reminder+COM-B Message intervention’s mechanisms of behavior change and avenues for improving its acceptability.

Our feasibility, acceptability, and engagement hypotheses were largely supported. Specifically, few AYAs were ineligible due to not owning a mobile device, we did not encounter major technical or AYA-reported problems with our electronic pill bottles or our low-cost text messaging approach, and our retention rate for enrolled participants was high. The majority of AYAs in the Reminder+COM-B Message intervention group reportedly read the messages or acknowledged receiving and noticing the messages (even if they did not read the content). Further, no AYAs in the Reminder+COM-B Message intervention group reported iatrogenic effects of the messages reducing their desire to take medicine. These findings suggest that using an electronic pill bottle to monitor adherence while receiving daily text messages with content designed to promote adherence is a feasible and acceptable intervention approach for AYAs with CKD taking daily antihypertensive medicine. Our qualitative results highlighted ways to improve message engagement, which may help enhance content interest for AYAs who reportedly did not read the majority of the Reminder+COM-B messages. Future researchers may consider adapting our approach to designing and testing messaging-based mHealth interventions for AYA medication adherence and incorporating objective engagement indices to bolster our subjective engagement findings from the current study.

Our Phase 2 pilot results showed that our primary outcome, daily postrandomization adherence, declined at a significantly faster rate in the Reminder-only Message active control group compared to the Reminder+COM-B Message intervention group. No significant group by time differences were observed for our secondary outcome, overall mean adherence, which likely reflected our sample size and statistical power for these analyses. Longitudinal research indicates that antihypertensive medication adherence is expected to decrease over time in AYAs with CKD [20], which was similarly observed in the Reminder-only Message active control group over the 4-week postrandomization phase. In contrast, the Reminder+COM-B Message intervention, which involved sending a bundled daily reminder message with an alternating capability or motivation message, appeared to stabilize daily adherence with some gradual improvements observed. Of note, during the first week of the postrandomization phase, the Reminder-only Message active control group demonstrated higher adherence than their baseline adherence and the Reminder-COM-B Message intervention group, which likely contributed to the significant group by time interaction observed during this monitoring period. It is possible that the Reminder-only Message active control group’s decline in adherence represented regression to their earlier baseline. Given that this was a pilot study, results of our pilot RCT should be interpreted with caution and understood as an important step in refining our new intervention and informing whether further testing is warranted, rather than evidence of its efficacy [32-34]. Our feasibility, acceptability, and preliminary Phase 2 results suggest that more rigorous future investigations of our Reminder+COM-B Message intervention could be a productive next step in developing efficacious mHealth approaches for improving AYA adherence.

There are several hypothesized reasons for why the Reminder+COM-B Message intervention group may have shown stable and gradually improved adherence during postrandomization. AYA qualitative interview results suggest the different daily Reminder+COM-B Message content may have been interesting to AYAs and helped them attend to messages as a cue to take their medicine. It is possible that the higher intensity of messaging compared to the Reminder-only Message active control group enhanced the impact on adherence. Further investigation with larger samples is needed to clarify the Reminder+COM-B Message intervention’s mechanistic effects on daily adherence in contrast to a daily simple reminder.

Unexpectedly, pre-post changes in AYAs’ perceptions of their adherence motivation and capability (survey responses) did not differ significantly by group and within-group changes were small in magnitude. The sample size was small, which limited statistical power to detect effects in these analyses. During qualitative interviews, some AYAs discussed how the Reminder+COM-B messages led to tangible behavioral and motivational changes that enhanced their adherence, but most viewed the message bundles as adherence cues. Considering the COM-B model upon which this intervention was designed [19], an alternative hypothesis is that the mechanism of change in daily adherence may be linked to improving *opportunity* (cue) to take medicine rather than changing AYAs’ perceptions of their adherence *capability* or *motivation*. This hypothesis is further supported by qualitative interviews suggesting that the Reminder+COM-B messages reminded AYAs to take their medicine regardless of message content. Further investigation is needed to elucidate how AYA perceptions of adherence capability and motivation can be modified via mHealth approaches to facilitate adherence behavior change.

In qualitative interviews about the Reminder+COM-B Message intervention, AYAs discussed suggested improvements to tailor message intensity, content, and timing to match adherence behavior and barriers, as well as AYA availability, receptivity, and engagement in the intervention. These major themes focused on behavioral components of the intervention and adherence and are suggestive of a just-in-time adaptive intervention (JITAI), which provides in-the-moment intervention exactly when AYAs need it most [35]. Adapting message intensity to match adherence (eg, send messages when medicine is missed) may enhance AYAs’ engagement by increasing message novelty, while more clearly demonstrating the link between intervention delivery and adherence behavior. Moving toward an adaptive format for message timing (eg, send messages when an AYA is available to read them) and content (eg, match message content to current barriers that could lead to nonadherence) may increase AYA receptivity to the intervention and enhance the likelihood that attending to message content will lead to adherence or implementing strategies to prevent nonadherence. Providing praise when medicine is taken may positively reinforce adherence behavior and enhance the probability that AYAs will take subsequent doses. These hypotheses await further testing in a modified version of our mHealth messaging intervention built within a JITAI framework.

### Limitations

The Reminder+COM-B messages were bundled and sent at the same frequency and in the same order to all AYAs randomized to this condition; hence, it is unknown how individual components (eg, motivation messages only), different message intensities (sending bundled vs single messages), or ordering may have differential effects on adherence. Future investigations that adhere to the Multiphase Optimization Strategy framework [36] to systemically and iteratively conduct optimization trials using novel experimental designs (eg, microrandomized trials [37]) may help identify specific intervention components delivered at particular times and intensities that maximize intervention acceptability and efficacy for an individual and form the basis for decision rules applied in a JITAI-based version of our intervention.

Our technology was limited in that we were unable to objectively evaluate AYA engagement by confirming if text messages were read. A key future direction is to incorporate objective engagement measures in evaluating our intervention approach. The sample size was small, which limited statistical power. However, we rigorously measured daily adherence data over 8 weeks, which yielded a larger number of data points for our analyses involving changes in daily adherence over time. AYAs were recruited from a single site and we monitored one prescribed antihypertensive medication per AYA, which may limit generalizability of the findings. AYAs were only offered electronic pill bottles, although some people use pillboxes to manage multiple medications. Researchers should consider offering the option of electronic pill bottles or pillboxes as a strategy for improving the acceptability and sustainability of objectively measuring daily adherence. Although we probed for major problems using the electronic pill bottles after Phase 2 and identified no issues in transmitting data from the bottles to our data collection portal, we were unable to personally observe every participant at each expected medication dose to obtain additional verification of adherence behavior. We included AYAs irrespective of their baseline adherence levels, which may have limited opportunities to observe adherence improvements. Further, we did not assess the impact of removing text messages on adherence to see if there was a lasting protective effect of text messaging on adherence, which should be examined in future studies.

We included AYAs within a larger age range; a more limited age range could provide greater insight into the Reminder+COM-B Message intervention’s acceptability within specific developmental phases (eg, young adulthood). A higher number of AYAs declined to enroll in the study due to lack of interest in participating. Although AYA enrollment challenges are common in behavioral intervention trials, our approach to introducing the study may benefit from adjustments to improve acceptability and engagement. Specifically, recruitment may benefit from deeper discussion of the altruistic reasons for study participation to enhance AYA interest [38], given that the primary reason for declining participation was lack of interest in research in general. Only AYAs randomized to the Reminder+COM-B Message intervention group completed qualitative interviews; future investigators should obtain AYA perspectives on receiving daily reminders only, as in the Reminder-only Message active control group (eg, to probe whether declines in adherence reflected fatigue from receiving the same messages). Additionally, the 8-week monitoring period limited the ability to examine associations between group allocation and clinical outcomes (eg, changes in estimated glomerular filtration rate), which should be evaluated in future studies designed to follow AYAs for longer periods of time.

### Conclusions

Our theoretically informed Reminder+COM-B mHealth messaging intervention appears to be feasible, acceptable, and promising for promoting objectively measured antihypertensive medication adherence in AYAs with CKD. Future research using similarly rigorous adherence outcome measures is needed to test refined versions of this intervention that incorporate AYA feedback and use study designs aimed at determining the most efficacious intervention components (eg, microrandomized trials [37]) to maximize the positive impact on AYAs’ medication adherence.

## Appendix

Multimedia Appendix 1 CONSORT-eHEALTH checklist (V 1.6.1).

## Abbreviations

AIC: Akaike information criterion
AMBS: Adolescent Medication Barriers Scale
AYA: adolescent/young adult
CKD: chronic kidney disease
JITAI: just-in-time adaptive intervention
mHealth: mobile health
NOE: Negative Outcome Expectancies
POE: Positive Outcome Expectancies
RCT: randomized controlled trial

## References

[1] Fathallah-Shaykh SA (2017). Proteinuria and progression of pediatric chronic kidney disease: lessons from recent clinical studies. Pediatr Nephrol.

[2] Wühl E, Schaefer F (2008). Therapeutic strategies to slow chronic kidney disease progression. Pediatr Nephrol.

[3] Pruette CS, Coburn SS, Eaton CK, Brady TM, Tuchman S, Mendley S, Fivush BA, Eakin MN, Riekert KA (2019). Does a multimethod approach improve identification of medication nonadherence in adolescents with chronic kidney disease?. Pediatr Nephrol.

[4] Hommel K, Ramsey R, Rich K, Ryan J, Roberts MC, Steele RG (2017). Adherence to pediatric treatment regimens. Handbook of pediatric psychology, 5th edition.

[5] Nieuwlaat R, Wilczynski N, Navarro T, Hobson N, Jeffery R, Keepanasseril A, Agoritsas T, Mistry N, Iorio A, Jack S, Sivaramalingam B, Iserman E, Mustafa RA, Jedraszewski D, Cotoi C, Haynes RB (2014). Interventions for enhancing medication adherence. Cochrane Database Syst Rev.

[6] (2018). Teens, social media & technology 2018. Pew Research Center.

[7] Lee Y, Cui Y, Tu M, Chen Y, Chang P (2018). Mobile Health to Maintain Continuity of Patient-Centered Care for Chronic Kidney Disease: Content Analysis of Apps. JMIR Mhealth Uhealth.

[8] Singh K, Diamantidis CJ, Ramani S, Bhavsar NA, Mara P, Warner J, Rodriguez J, Wang T, Wright-Nunes J (2019). Patients' and Nephrologists' Evaluation of Patient-Facing Smartphone Apps for CKD. Clin J Am Soc Nephrol.

[9] Nguyen E, Bugno L, Kandah C, Plevinsky J, Poulopoulos N, Wojtowicz A, Schneider KL, Greenley RN (2016). Is There a Good App for That? Evaluating m-Health Apps for Strategies That Promote Pediatric Medication Adherence. Telemed J E Health.

[10] Kosse RC, Bouvy ML, de Vries TW, Koster ES (2019). Effect of a mHealth intervention on adherence in adolescents with asthma: A randomized controlled trial. Respir Med.

[11] Ramsey RR, Holbein CE, Powers SW, Hershey AD, Kabbouche MA, O'Brien HL, Kacperski J, Shepard J, Hommel KA (2018). A pilot investigation of a mobile phone application and progressive reminder system to improve adherence to daily prevention treatment in adolescents and young adults with migraine. Cephalalgia.

[12] Fenerty SD, West C, Davis SA, Kaplan SG, Feldman SR (2012). The effect of reminder systems on patients' adherence to treatment. Patient Prefer Adherence.

[13] Vervloet M, Linn AJ, van Weert JCM, de Bakker DH, Bouvy ML, van Dijk L (2012). The effectiveness of interventions using electronic reminders to improve adherence to chronic medication: a systematic review of the literature. J Am Med Inform Assoc.

[14] Anderson LM, Leonard S, Jonassaint J, Lunyera J, Bonner M, Shah N (2018). Mobile health intervention for youth with sickle cell disease: Impact on adherence, disease knowledge, and quality of life. Pediatr Blood Cancer.

[15] Creary S, Chisolm D, Stanek J, Hankins J, O'Brien SH (2019). A Multidimensional Electronic Hydroxyurea Adherence Intervention for Children With Sickle Cell Disease: Single-Arm Before-After Study. JMIR Mhealth Uhealth.

[16] Zhang S, Hamburger E, Kahanda S, Lyttle M, Williams R, Jaser SS (2018). Engagement with a Text-Messaging Intervention Improves Adherence in Adolescents with Type 1 Diabetes: Brief Report. Diabetes Technol Ther.

[17] Badawy SM, Barrera L, Sinno MG, Kaviany S, O'Dwyer LC, Kuhns LM (2017). Text Messaging and Mobile Phone Apps as Interventions to Improve Adherence in Adolescents With Chronic Health Conditions: A Systematic Review. JMIR Mhealth Uhealth.

[18] Badawy SM, Thompson AA, Kuhns LM (2017). Medication Adherence and Technology-Based Interventions for Adolescents With Chronic Health Conditions: A Few Key Considerations. JMIR Mhealth Uhealth.

[19] Michie S, van Stralen MM, West R (2011). The behaviour change wheel: a new method for characterising and designing behaviour change interventions. Implement Sci.

[20] Eaton CK, Eakin MN, Coburn S, Pruette CS, Brady TM, Fivush BA, Mendley S, Tuchman S, Riekert KA (2019). Patient Health Beliefs and Characteristics Predict Longitudinal Antihypertensive Medication Adherence in Adolescents With CKD. J Pediatr Psychol.

[21] Latimer AE, Rench TA, Rivers SE, Katulak NA, Materese SA, Cadmus L, Hicks A, Keany Hodorowski J, Salovey P (2008). Promoting participation in physical activity using framed messages: an application of prospect theory. Br J Health Psychol.

[22] Gallagher KM, Updegraff JA (2012). Health message framing effects on attitudes, intentions, and behavior: a meta-analytic review. Ann Behav Med.

[23] Noar SM (2006). A 10-year retrospective of research in health mass media campaigns: where do we go from here?. J Health Commun.

[24] Zhao X, Villagran MM, Kreps GL, McHorney C (2012). Gain versus loss framing in adherence-promoting communication targeting patients with chronic diseases: the moderating effect of individual time perspective. Health Commun.

[25] Cho H, Salmon C (2007). Unintended effects of health communication campaigns. J Commun.

[26] Simons LE, Blount RL (2007). Identifying barriers to medication adherence in adolescent transplant recipients. J Pediatr Psychol.

[27] Riekert K, Drotar D (2002). The Beliefs About Medications Scale: Development, reliability, and validity. J Clin Psychol Med Settings.

[28] Riekert K, Rand C (2002). Electronic monitoring of medication adherence: When is high-tech best? J Clin Psychol Med S. J Clin Psychol Med S.

[29] Riekert K (2000). Health beliefs and medication adherence among adolescents with chronic health conditions. Doctoral dissertation, Case Western Reserve University, Cleveland, OH.

[30] Eakin MN, Chung S, Hoehn J, Borrelli B, Rand-Giovannetti D, Riekert KA (2017). Development and validation of CF-Medication Beliefs Questionnaire: A mixed-methods approach. J Cyst Fibros.

[31] Hilbe J (2011). Negative binomial regression. 2nd ed.

[32] Leon AC, Davis LL, Kraemer HC (2011). The role and interpretation of pilot studies in clinical research. J Psychiatr Res.

[33] Bowen DJ, Kreuter M, Spring B, Cofta-Woerpel L, Linnan L, Weiner D, Bakken S, Kaplan CP, Squiers L, Fabrizio C, Fernandez M (2009). How we design feasibility studies. Am J Prev Med.

[34] Kistin C, Silverstein M (2015). Pilot Studies: A Critical but Potentially Misused Component of Interventional Research. JAMA.

[35] Nahum-Shani I, Smith SN, Spring BJ, Collins LM, Witkiewitz K, Tewari A, Murphy SA (2018). Just-in-Time Adaptive Interventions (JITAIs) in Mobile Health: Key Components and Design Principles for Ongoing Health Behavior Support. Ann Behav Med.

[36] Collins LM, Murphy SA, Strecher V (2007). The multiphase optimization strategy (MOST) and the sequential multiple assignment randomized trial (SMART): new methods for more potent eHealth interventions. Am J Prev Med.

[37] Klasnja P, Hekler EB, Shiffman S, Boruvka A, Almirall D, Tewari A, Murphy SA (2015). Microrandomized trials: An experimental design for developing just-in-time adaptive interventions. Health Psychol.

[38] Hendricks-Ferguson VL, Cherven BO, Burns DS, Docherty SL, Phillips-Salimi CR, Roll L, Stegenga KA, Donovan Stickler M, Haase JE (2013). Recruitment strategies and rates of a multi-site behavioral intervention for adolescents and young adults with cancer. J Pediatr Health Care.

