# Supplementary material for: Text Messaging Adherence Intervention for Adolescents and Young Adults with Chronic Kidney Disease: Pilot Randomized Controlled Trial and Stakeholder Interviews
Source: J Med Internet Res. 2020 Aug 14;22(8):e19861. doi: 10.2196/19861 (PMC7455868; doi:10.2196/19861)
Supplement: Multimedia Appendix 1 [file jmir_v22i8e19861_app1.pdf]

CONSORT-EHEALTH (V 1.6.1) - Submission/Publication Form

The CONSORT-EHEALTH checklist is intended for authors of randomized trials evaluating web-based and Internet-based applications/interventions, including mobile interventions, electronic games (incl multiplayer games), social media, certain telehealth applications, and other interactive and/or networked electronic applications. Some of the items (e.g. all subitems under item 5 - description of the intervention) may also be applicable for other study designs.

The goal of the CONSORT EHEALTH checklist and guideline is to be
a) a guide for reporting for authors of RCTs,
b) to form a basis for appraisal of an ehealth trial (in terms of validity)

CONSORT-EHEALTH items/subitems are MANDATORY reporting items for studies published in the Journal of Medical Internet Research and other journals / scientific societies endorsing the checklist.

Items numbered 1., 2., 3., 4a., 4b etc are original CONSORT or CONSORT-NPT (non-pharmacologic treatment) items.
Items with Roman numerals (i., ii, iii, iv etc.) are CONSORT-EHEALTH extensions/clarifications.

As the CONSORT-EHEALTH checklist is still considered in a formative stage, we would ask that you also RATE ON A SCALE OF 1-5 how important/useful you feel each item is FOR THE PURPOSE OF THE CHECKLIST and reporting guideline (optional).

Mandatory reporting items are marked with a red *.
In the textboxes, either copy & paste the relevant sections from your manuscript into this form - please include any quotes from your manuscript in QUOTATION MARKS,
or answer directly by providing additional information not in the manuscript, or elaborating on why the item was not relevant for this study.

YOUR ANSWERS WILL BE PUBLISHED AS A SUPPLEMENTARY FILE TO YOUR PUBLICATION IN JMIR AND ARE CONSIDERED PART OF YOUR PUBLICATION (IF ACCEPTED).
Please fill in these questions diligently. Information will not be copyedited, so please use proper spelling and grammar, use correct capitalization, and avoid abbreviations.

DO NOT FORGET TO SAVE AS PDF _AND_ CLICK THE SUBMIT BUTTON SO YOUR ANSWERS ARE IN OUR DATABASE !!!

Citation Suggestion (if you append the pdf as Appendix we suggest to cite this paper in the caption):
Eysenbach G, CONSORT-EHEALTH Group
CONSORT-EHEALTH: Improving and Standardizing Evaluation Reports of Web-based and

Mobile Health Interventions
J Med Internet Res 2011;13(4):e126
URL: http://www.jmir.org/2011/4/e126/
doi: 10.2196/jmir.1923
PMID: 22209829

* Required

Your name *

First Last

Cyd Eaton

Primary Affiliation (short), City, Country *

University of Toronto, Toronto, Canada

Johns Hopkins School of Medicine, Baltimore,

Your e-mail address *

abc@gmail.com

ceaton4@jhmi.edu

Title of your manuscript *

Provide the (draft) title of your manuscript.

A Mobile Health Messaging Intervention for Adherence in AYAs with Chronic Kidney Disease: Results of a Pilot RCT and Stakeholder Interviews

Name of your App/Software/Intervention *

If there is a short and a long/alternate name, write the short name first and add the long name in brackets.

Reminder+COM-B Message Intervention

Evaluated Version (if any)

e.g. "V1", "Release 2017-03-01", "Version 2.0.27913"

Version 1

Language(s) *

What language is the intervention/app in? If multiple languages are available, separate by comma (e.g. "English, French")

English

URL of your Intervention Website or App

e.g. a direct link to the mobile app on app in appstore (itunes, Google Play), or URL of the website. If the intervention is a DVD or hardware, you can also link to an Amazon page.

Your answer

URL of an image/screenshot (optional)

Your answer

Accessibility *

Can an enduser access the intervention presently?

- ○ access is free and open
- ○ access only for special usergroups, not open
- ○ access is open to everyone, but requires payment/subscription/in-app purchases
- ◉ app/intervention no longer accessible
- ○ Other:

Primary Medical Indication/Disease/Condition *

e.g. "Stress", "Diabetes", or define the target group in brackets after the condition, e.g. "Autism (Parents of children with)", "Alzheimers (Informal Caregivers of)"

chronic kidney disease (adolescents and youn

Primary Outcomes measured in trial *

comma-separated list of primary outcomes reported in the trial

objective antihypertensive medication adherer

Secondary/other outcomes

Are there any other outcomes the intervention is expected to affect?

Participant surveys of adherence self-efficacy, adherence barriers, outcome expectancies for taking medicine, and motivation for and importance of taking medicine

Recommended "Dose" *

What do the instructions for users say on how often the app should be used?

- ( ) Approximately Daily
- ( ) Approximately Weekly
- ( ) Approximately Monthly
- ( ) Approximately Yearly
- ( ) "as needed"
- (•) Other: The intervention involved sending daily text messages to users' mobile phones

Approx. Percentage of Users (starters) still using the app as recommended after 3 months *

- ( ) unknown / not evaluated
- ( ) 0-10%
- ( ) 11-20%
- ( ) 21-30%
- ( ) 31-40%
- ( ) 41-50%
- ( ) 51-60%
- ( ) 61-70%
- ( ) 71%-80%
- ( ) 81-90%
- ( ) 91-100%
- (•) Other: The trial lasted 8 weeks with 4 weeks of receiving text messages. After the stu

Overall, was the app/intervention effective? *

- (selected) yes: all primary outcomes were significantly better in intervention group vs control
- partly: SOME primary outcomes were significantly better in intervention group vs control
- no statistically significant difference between control and intervention
- potentially harmful: control was significantly better than intervention in one or more outcomes
- inconclusive: more research is needed
- Other:

Article Preparation Status/Stage *

At which stage in your article preparation are you currently (at the time you fill in this form)

- not submitted yet - in early draft status
- not submitted yet - in late draft status, just before submission
- (selected) submitted to a journal but not reviewed yet
- submitted to a journal and after receiving initial reviewer comments
- submitted to a journal and accepted, but not published yet
- published
- Other:

Journal *

If you already know where you will submit this paper (or if it is already submitted), please provide the journal name (if it is not JMIR, provide the journal name under "other")

- ○ not submitted yet / unclear where I will submit this
- ◉ Journal of Medical Internet Research (JMIR)
- ○ JMIR mHealth and UHealth
- ○ JMIR Serious Games
- ○ JMIR Mental Health
- ○ JMIR Public Health
- ○ JMIR Formative Research
- ○ Other JMIR sister journal
- ○ Other:

Is this a full powered effectiveness trial or a pilot/feasibility trial? *

- ◉ Pilot/feasibility
- ○ Fully powered

Manuscript tracking number *

If this is a JMIR submission, please provide the manuscript tracking number under "other" (The ms tracking number can be found in the submission acknowledgement email, or when you login as author in JMIR. If the paper is already published in JMIR, then the ms tracking number is the four-digit number at the end of the DOI, to be found at the bottom of each published article in JMIR)

- ○ no ms number (yet) / not (yet) submitted to / published in JMIR
- ◉ Other: 19861

TITLE AND ABSTRACT

1a) TITLE: Identification as a randomized trial in the title

1a) Does your paper address CONSORT item 1a? *

I.e does the title contain the phrase "Randomized Controlled Trial"? (if not, explain the reason under "other")

- (•) yes
- ( ) Other:

1a-i) Identify the mode of delivery in the title

Identify the mode of delivery. Preferably use “web-based” and/or “mobile” and/or “electronic game” in the title. Avoid ambiguous terms like “online”, “virtual”, “interactive”. Use “Internet-based” only if Intervention includes non-web-based Internet components (e.g. email), use “computer-based” or “electronic” only if offline products are used. Use “virtual” only in the context of “virtual reality” (3-D worlds). Use “online” only in the context of “online support groups”. Complement or substitute product names with broader terms for the class of products (such as “mobile” or “smart phone” instead of “iphone”), especially if the application runs on different platforms.

	1	2	3	4	5	
subitem not at all important	( )	( )	( )	(•)	( )	essential

Does your paper address subitem 1a-i? *

Copy and paste relevant sections from manuscript title (include quotes in quotation marks "like this" to indicate direct quotes from your manuscript), or elaborate on this item by providing additional information not in the ms, or briefly explain why the item is not applicable/relevant for your study

"A Mobile Health Messaging Intervention"

1a-ii) Non-web-based components or important co-interventions in title

Mention non-web-based components or important co-interventions in title, if any (e.g., “with telephone support”).

	1	2	3	4	5	
subitem not at all important	○	○	●	○	○	essential

Does your paper address subitem 1a-ii?

Copy and paste relevant sections from manuscript title (include quotes in quotation marks "like this" to indicate direct quotes from your manuscript), or elaborate on this item by providing additional information not in the ms, or briefly explain why the item is not applicable/relevant for your study

N/A: This study does not contain a co-intervention or non-mHealth components

1a-iii) Primary condition or target group in the title

Mention primary condition or target group in the title, if any (e.g., “for children with Type I Diabetes”) Example: A Web-based and Mobile Intervention with Telephone Support for Children with Type I Diabetes: Randomized Controlled Trial

	1	2	3	4	5	
subitem not at all important	○	○	○	○	●	essential

Does your paper address subitem 1a-iii? *

Copy and paste relevant sections from manuscript title (include quotes in quotation marks "like this" to indicate direct quotes from your manuscript), or elaborate on this item by providing additional information not in the ms, or briefly explain why the item is not applicable/relevant for your study

"AYAs with Chronic Kidney Disease"

1b) ABSTRACT: Structured summary of trial design, methods, results, and conclusions

NPT extension: Description of experimental treatment, comparator, care providers, centers, and blinding status.

1b-i) Key features/functionalities/components of the intervention and comparator in the METHODS section of the ABSTRACT

Mention key features/functionalities/components of the intervention and comparator in the abstract. If possible, also mention theories and principles used for designing the site. Keep in mind the needs of systematic reviewers and indexers by including important synonyms. (Note: Only report in the abstract what the main paper is reporting. If this information is missing from the main body of text, consider adding it)

	1	2	3	4	5	
subitem not at all important	○	○	○	○	◉	essential

Does your paper address subitem 1b-i? *

Copy and paste relevant sections from the manuscript abstract (include quotes in quotation marks "like this" to indicate direct quotes from your manuscript), or elaborate on this item by providing additional information not in the ms, or briefly explain why the item is not applicable/relevant for your study

"This multi-phase investigation developed and tested a theoretically-informed mHealth messaging intervention (based on the COM-B model for generating behavior change) to improve antihypertensive medication adherence in AYAs with CKD in a pilot randomized controlled trial." "In Phase 2, the Reminder+COM-B message intervention was tested against a Reminder-only message active control condition in an 8-week pilot randomized controlled trial."

1b-ii) Level of human involvement in the METHODS section of the ABSTRACT

Clarify the level of human involvement in the abstract, e.g., use phrases like “fully automated” vs. “therapist/nurse/care provider/physician-assisted” (mention number and expertise of providers involved, if any). (Note: Only report in the abstract what the main paper is reporting. If this information is missing from the main body of text, consider adding it)

	1	2	3	4	5	
subitem not at all important	○	◉	○	○	○	essential

Does your paper address subitem 1b-ii?

Copy and paste relevant sections from the manuscript abstract (include quotes in quotation marks "like this" to indicate direct quotes from your manuscript), or elaborate on this item by providing additional information not in the ms, or briefly explain why the item is not applicable/relevant for your study

This detail is discussed in the Method section

1b-iii) Open vs. closed, web-based (self-assessment) vs. face-to-face assessments in the METHODS section of the ABSTRACT

Mention how participants were recruited (online vs. offline), e.g., from an open access website or from a clinic or a closed online user group (closed usergroup trial), and clarify if this was a purely web-based trial, or there were face-to-face components (as part of the intervention or for assessment). Clearly say if outcomes were self-assessed through questionnaires (as common in web-based trials). Note: In traditional offline trials, an open trial (open-label trial) is a type of clinical trial in which both the researchers and participants know which treatment is being administered. To avoid confusion, use “blinded” or “unblinded” to indicated the level of blinding instead of “open”, as “open” in web-based trials usually refers to “open access” (i.e. participants can self-enrol). (Note: Only report in the abstract what the main paper is reporting. If this information is missing from the main body of text, consider adding it)

	1	2	3	4	5	
subitem not at all important	○	◉	○	○	○	essential

Does your paper address subitem 1b-iii?

Copy and paste relevant sections from the manuscript abstract (include quotes in quotation marks "like this" to indicate direct quotes from your manuscript), or elaborate on this item by providing additional information not in the ms, or briefly explain why the item is not applicable/relevant for your study

This detail is discussed in the Method section

1b-iv) RESULTS section in abstract must contain use data

Report number of participants enrolled/assessed in each group, the use/uptake of the intervention (e.g., attrition/adherence metrics, use over time, number of logins etc.), in addition to primary/secondary outcomes. (Note: Only report in the abstract what the main paper is reporting. If this information is missing from the main body of text, consider adding it)

	1	2	3	4	5	
subitem not at all important	○	○	○	◉	○	essential

Does your paper address subitem 1b-iv?

Copy and paste relevant sections from the manuscript abstract (include quotes in quotation marks "like this" to indicate direct quotes from your manuscript), or elaborate on this item by providing additional information not in the ms, or briefly explain why the item is not applicable/relevant for your study

Following Phase 1, 34 AYAs with CKD (Mean age=16.59 years, 41% female, 38% African American/Black, 35% hypertension diagnosis; Reminder+COM-B message intervention message group=18 AYAs, Reminder-only message active control group=16 AYAs) completed the Phase 2 pilot randomized controlled trial. All AYAs in the Reminder+COM-B message intervention group completed a Phase 3 qualitative interview. Overall, study procedures were feasible to implement and 0/18 AYAs in the Reminder+COM-B message intervention group reported the messages reduced their desire to take medicine. Pre-randomization, there were no significant group differences in the rate of change in daily adherence over time. However, post-randomization, there was a significant group by time interaction (B=.01, P=.04) in which daily adherence decreased significantly over time in the Reminder-only active control group but remained stable in the Reminder+COM-B message intervention group.

1b-v) CONCLUSIONS/DISCUSSION in abstract for negative trials

Conclusions/Discussions in abstract for negative trials: Discuss the primary outcome - if the trial is negative (primary outcome not changed), and the intervention was not used, discuss whether negative results are attributable to lack of uptake and discuss reasons. (Note: Only report in the abstract what the main paper is reporting. If this information is missing from the main body of text, consider adding it)

	1	2	3	4	5	
subitem not at all important	○	○	○	◉	○	essential

Does your paper address subitem 1b-v?

Copy and paste relevant sections from the manuscript abstract (include quotes in quotation marks "like this" to indicate direct quotes from your manuscript), or elaborate on this item by providing additional information not in the ms, or briefly explain why the item is not applicable/relevant for your study

N/A--our results for the intervention tested were promising beyond the Reminder-only active control group's simple reminder condition, which we discuss in the Conclusions section.

INTRODUCTION

2a) In INTRODUCTION: Scientific background and explanation of rationale

2a-i) Problem and the type of system/solution

Describe the problem and the type of system/solution that is object of the study: intended as stand-alone intervention vs. incorporated in broader health care program? Intended for a particular patient population? Goals of the intervention, e.g., being more cost-effective to other interventions, replace or complement other solutions? (Note: Details about the intervention are provided in “Methods” under 5)

	1	2	3	4	5	
subitem not at all important	○	○	○	○	◉	essential

Does your paper address subitem 2a-i? *

Copy and paste relevant sections from the manuscript (include quotes in quotation marks "like this" to indicate direct quotes from your manuscript), or elaborate on this item by providing additional information not in the ms, or briefly explain why the item is not applicable/relevant for your study

"The current study aimed to address limitations of prior interventions by developing and testing a theoretically-informed antihypertensive medication adherence-promoting mHealth messaging intervention for AYAs with CKD using objective adherence outcomes and qualitative stakeholder feedback." "We report the results of this mixed methods investigation, which involved (1) developing the Reminder+COM-B message intervention for antihypertensive medication adherence in AYAs with CKD (Phase 1), (2) preliminarily evaluating the Reminder+COM-B message intervention against a Reminder-only message active control condition in an 8-week pilot randomized controlled trial (RCT; Phase 2), and (3) obtaining post-study qualitative feedback from AYAs randomized to the Reminder+COM-B message intervention (Phase 3)."

2a-ii) Scientific background, rationale: What is known about the (type of) system

Scientific background, rationale: What is known about the (type of) system that is the object of the study (be sure to discuss the use of similar systems for other conditions/diagnoses, if appropiate), motivation for the study, i.e. what are the reasons for and what is the context for this specific study, from which stakeholder viewpoint is the study performed, potential impact of findings [2]. Briefly justify the choice of the comparator.

	1	2	3	4	5	
subitem not at all important	○	○	○	○	◉	essential

Does your paper address subitem 2a-ii? *

Copy and paste relevant sections from the manuscript (include quotes in quotation marks "like this" to indicate direct quotes from your manuscript), or elaborate on this item by providing additional information not in the ms, or briefly explain why the item is not applicable/relevant for your study

See paragraph beginning: "mHealth interventions have been developed for medication adherence in children and AYAs with other medical conditions (e.g., sickle cell disease, asthma, type 1 diabetes, migraine)." Rationale for our comparator condition: "However, these interventions have primarily relied on daily dose reminders [9-11], which show short-term effects on adherence and have barriers to long-term practicality (e.g., repeated reminders could be viewed as intrusive instead of helpful) [12,13]...Opportunity is often targeted in mHealth interventions via medicine reminder messages." Motivation for current study: "The current study aimed to address limitations of prior interventions by developing and testing a theoretically-informed antihypertensive medication adherence-promoting mHealth messaging intervention for AYAs with CKD using objective adherence outcomes and qualitative stakeholder feedback."

2b) In INTRODUCTION: Specific objectives or hypotheses

Does your paper address CONSORT subitem 2b? *

Copy and paste relevant sections from the manuscript (include quotes in quotation marks "like this" to indicate direct quotes from your manuscript), or elaborate on this item by providing additional information not in the ms, or briefly explain why the item is not applicable/relevant for your study

"We report the results of this mixed methods investigation, which involved (1) developing the Reminder+COM-B message intervention for antihypertensive medication adherence in AYAs with CKD (Phase 1), (2) preliminarily evaluating the Reminder+COM-B message intervention against a Reminder-only message active control condition in an 8-week pilot randomized controlled trial (RCT; Phase 2), and (3) obtaining post-study qualitative feedback from AYAs randomized to the Reminder+COM-B message intervention (Phase 3). We hypothesized that (a) study procedures would be feasible and acceptable, (b) post-randomization (Phase 2), daily adherence (dose taken or not) would show a faster rate of improvement in the Reminder+COM-B message intervention group compared to the Reminder-only active control group, (c) pre-post survey scores representing AYA perceptions of adherence motivation and capability would show greater improvement in the Reminder+COM-B message intervention group compared to the Reminder-only active control group (Phase 2), and (d) <10% of AYAs would report that the Reminder+COM-B message intervention reduced their desire to take medicine (Phase 3). Given conventions for analyzing adherence as a mean, we also examined mean changes in adherence from baseline to post-randomization by group allocation (Phase 2). During qualitative interviews (Phase 3), we probed AYAs' perceptions on the Reminder+COM-B message intervention's mechanisms of behavior change and suggestions for improving its efficacy."

METHODS

3a) Description of trial design (such as parallel, factorial) including allocation ratio

Does your paper address CONSORT subitem 3a? *

Copy and paste relevant sections from the manuscript (include quotes in quotation marks "like this" to indicate direct quotes from your manuscript), or elaborate on this item by providing additional information not in the ms, or briefly explain why the item is not applicable/relevant for your study

"The first 4 weeks of the pilot RCT (baseline) evaluated AYA adherence without sending any messages. AYAs were randomized to the Reminder+COM-B message intervention or Reminder-only message active control group and received their respective group’s messages for the last 4 weeks of the pilot RCT. AYAs were randomized in a 1:1 basis to either group. A random number generator was used for simple randomization and AYAs were assigned consecutively to a group. Blinding the study team to group assignment was not possible; however, the messages were delivered remotely via text message rather than face-to-face."

3b) Important changes to methods after trial commencement (such as eligibility criteria), with reasons

Does your paper address CONSORT subitem 3b? *

Copy and paste relevant sections from the manuscript (include quotes in quotation marks "like this" to indicate direct quotes from your manuscript), or elaborate on this item by providing additional information not in the ms, or briefly explain why the item is not applicable/relevant for your study

N/A--no changes were made to the trial design after the study began

3b-i) Bug fixes, Downtimes, Content Changes

Bug fixes, Downtimes, Content Changes: ehealth systems are often dynamic systems. A description of changes to methods therefore also includes important changes made on the intervention or comparator during the trial (e.g., major bug fixes or changes in the functionality or content) (5-iii) and other “unexpected events” that may have influenced study design such as staff changes, system failures/downtimes, etc. [2].

	1	2	3	4	5	
subitem not at all important	○	○	○	◉	○	essential

Does your paper address subitem 3b-i?

Copy and paste relevant sections from the manuscript (include quotes in quotation marks "like this" to indicate direct quotes from your manuscript), or elaborate on this item by providing additional information not in the ms, or briefly explain why the item is not applicable/relevant for your study

N/A--no changes to the ehealth system were made after the study began

4a) Eligibility criteria for participants

Does your paper address CONSORT subitem 4a? *

Copy and paste relevant sections from the manuscript (include quotes in quotation marks "like this" to indicate direct quotes from your manuscript), or elaborate on this item by providing additional information not in the ms, or briefly explain why the item is not applicable/relevant for your study

"Recruitment. The Johns Hopkins School of Medicine Institutional Review Board approved all study procedures prior to recruitment, which occurred at a single pediatric nephrology clinic (October 2018-November 2019). Trained study staff identified potentially eligible AYAs via clinic roster and electronic medical record review. Inclusion criteria were: ages 11-21 years old, CKD diagnosis, current antihypertensive medication prescription (pill form only), and access to a mobile phone that received text messages. Exclusion criteria were: underwent solid organ transplantation, received dialysis, had a sibling participating in the study, unable to understand spoken English, had a developmental delay or significant cognitive impairment precluding their ability to complete study procedures, or declined to use the electronic adherence monitoring device or be audio-recorded during qualitative interviews. Potentially eligible AYAs were mailed or emailed a letter describing the study and providing the opportunity to opt out of recruitment. AYAs who did not opt out of were contacted by telephone to provide study details, conduct further eligibility screening, and, if eligible and interested in enrolling, coordinate informed consent/assent procedures. Informed consent/assent procedures were conducted with participants during a telephone call with study staff (AYAs >18 years old provided consent for themselves; for AYAs <18 years old, a primary caregiver provided informed consent for their child and the AYA provided informed assent for themselves)."

4a-i) Computer / Internet literacy

Computer / Internet literacy is often an implicit “de facto” eligibility criterion - this should be explicitly clarified.

	1	2	3	4	5	
subitem not at all important	○	◉	○	○	○	essential

Does your paper address subitem 4a-i?

Copy and paste relevant sections from the manuscript (include quotes in quotation marks "like this" to indicate direct quotes from your manuscript), or elaborate on this item by providing additional information not in the ms, or briefly explain why the item is not applicable/relevant for your study

Eligibility criteria included: "access to a mobile phone that received text messages."

4a-ii) Open vs. closed, web-based vs. face-to-face assessments:

Open vs. closed, web-based vs. face-to-face assessments: Mention how participants were recruited (online vs. offline), e.g., from an open access website or from a clinic, and clarify if this was a purely web-based trial, or there were face-to-face components (as part of the intervention or for assessment), i.e., to what degree got the study team to know the participant. In online-only trials, clarify if participants were quasi-anonymous and whether having multiple identities was possible or whether technical or logistical measures (e.g., cookies, email confirmation, phone calls) were used to detect/prevent these.

	1	2	3	4	5	
subitem not at all important	○	○	○	◉	○	essential

Does your paper address subitem 4a-ii? *

Copy and paste relevant sections from the manuscript (include quotes in quotation marks "like this" to indicate direct quotes from your manuscript), or elaborate on this item by providing additional information not in the ms, or briefly explain why the item is not applicable/relevant for your study

Recruitment: "Potentially eligible AYAs were mailed or emailed a letter describing the study and providing the opportunity to opt out of recruitment. AYAs who did not opt out of were contacted by telephone to provide study details, conduct further eligibility screening, and, if eligible and interested in enrolling, coordinate informed consent/assent procedures. Informed consent/assent procedures were conducted with participants during a telephone call with study staff (AYAs >18 years old provided consent for themselves; for AYAs <18 years old, a primary caregiver provided informed consent for their child and the AYA provided informed assent for themselves)." Phase 1: "The initial intervention message pool was presented to 10 AYAs with CKD (M age=16.50 years, SD=3.41, range=12-21 years) during a semi-structured telephone interview (~60 minutes; audio-recorded for this study)." Phase 2: "Phase 2: Pilot RCT. AYAs were mailed electronic pill bottles for monitoring adherence (see Objective Medication Adherence section). After the bottle was delivered, study staff conducted a telephone call with the AYA to describe how the bottle worked, transfer monitored antihypertensive medicine into the bottle, and answer AYA or caregiver questions. AYAs were instructed to use the study bottle for their antihypertensive medicine during the 8-week pilot RCT and transfer any refills to the study bottle during this period. AYAs were informed that the study text messages would be sent to their mobile phone at some point during the pilot RCT but were not provided with a specific date. Text messages containing the Reminder+COM-B message intervention or Reminder-only active control messages were sent from a single study number via REDCap's Twilio interface. A brief demographic survey was sent to AYAs ≥18 years old and caregivers of AYAs <18 years old via REDCap. AYAs completed online surveys (Qualtrics) at baseline and after the pilot RCT ended." Phase 3: Phase 3: Qualitative Interviews. Following completion of Phase 2, AYAs in the Reminder+COM-B message intervention group were invited to complete audio-recorded and transcribed qualitative interviews (~30 minutes) by telephone."

4a-iii) Information giving during recruitment

Information given during recruitment. Specify how participants were briefed for recruitment and in the informed consent procedures (e.g., publish the informed consent documentation as appendix, see also item X26), as this information may have an effect on user self-selection, user expectation and may also bias results.

	1	2	3	4	5	
subitem not at all important	○	○	●	○	○	essential

Does your paper address subitem 4a-iii?

Copy and paste relevant sections from the manuscript (include quotes in quotation marks "like this" to indicate direct quotes from your manuscript), or elaborate on this item by providing additional information not in the ms, or briefly explain why the item is not applicable/relevant for your study

"Potentially eligible AYAs were mailed or emailed a letter describing the study and providing the opportunity to opt out of recruitment. AYAs who did not opt out of were contacted by telephone to provide study details, conduct further eligibility screening, and, if eligible and interested in enrolling, coordinate informed consent/assent procedures. Informed consent/assent procedures were conducted with participants during a telephone call with study staff (AYAs >18 years old provided consent for themselves; for AYAs <18 years old, a primary caregiver provided informed consent for their child and the AYA provided informed assent for themselves)."

4b) Settings and locations where the data were collected

Does your paper address CONSORT subitem 4b? *

Copy and paste relevant sections from the manuscript (include quotes in quotation marks "like this" to indicate direct quotes from your manuscript), or elaborate on this item by providing additional information not in the ms, or briefly explain why the item is not applicable/relevant for your study

Phase 1: "The initial intervention message pool was presented to 10 AYAs with CKD (M age=16.50 years, SD=3.41, range=12-21 years) during a semi-structured telephone interview (~60 minutes; audio-recorded for this study)." Phase 2: "A brief demographic survey was sent to AYAs ≥18 years old and caregivers of AYAs <18 years old via REDCap. AYAs completed online surveys (Qualtrics) at baseline and after the pilot RCT ended...Objective Medication Adherence. AdhereTech bottles electronically-assessed daily antihypertensive medication adherence via a cellular-connected cap that recorded the date and time when the bottle cap was opened and closed." Phase 3: "Phase 3: Qualitative Interviews. Following completion of Phase 2, AYAs in the Reminder+COM-B message intervention group were invited to complete audio-recorded and transcribed qualitative interviews (~30 minutes) by telephone."

4b-i) Report if outcomes were (self-)assessed through online questionnaires

Clearly report if outcomes were (self-)assessed through online questionnaires (as common in web-based trials) or otherwise.

	1	2	3	4	5	
subitem not at all important	○	○	○	○	◉	essential

Does your paper address subitem 4b-i? *

Copy and paste relevant sections from the manuscript (include quotes in quotation marks "like this" to indicate direct quotes from your manuscript), or elaborate on this item by providing additional information not in the ms, or briefly explain why the item is not applicable/relevant for your study

Phase 1: "The initial intervention message pool was presented to 10 AYAs with CKD (M age=16.50 years, SD=3.41, range=12-21 years) during a semi-structured telephone interview (~60 minutes; audio-recorded for this study)." Phase 2: "A brief demographic survey was sent to AYAs ≥18 years old and caregivers of AYAs <18 years old via REDCap. AYAs completed online surveys (Qualtrics) at baseline and after the pilot RCT ended...Objective Medication Adherence. AdhereTech bottles electronically-assessed daily antihypertensive medication adherence via a cellular-connected cap that recorded the date and time when the bottle cap was opened and closed." Phase 3: "Phase 3: Qualitative Interviews. Following completion of Phase 2, AYAs in the Reminder+COM-B message intervention group were invited to complete audio-recorded and transcribed qualitative interviews (~30 minutes) by telephone."

4b-ii) Report how institutional affiliations are displayed

Report how institutional affiliations are displayed to potential participants [on ehealth media], as affiliations with prestigious hospitals or universities may affect volunteer rates, use, and reactions with regards to an intervention.(Not a required item – describe only if this may bias results)

	1	2	3	4	5	
subitem not at all important	○	◉	○	○	○	essential

Does your paper address subitem 4b-ii?

Copy and paste relevant sections from the manuscript (include quotes in quotation marks "like this" to indicate direct quotes from your manuscript), or elaborate on this item by providing additional information not in the ms, or briefly explain why the item is not applicable/relevant for your study

N/A--all participants were current patients at our recruitment site. The text messaging intervention did not contain the institution name.

5) The interventions for each group with sufficient details to allow replication, including how and when they were actually administered

5-i) Mention names, credential, affiliations of the developers, sponsors, and owners

Mention names, credential, affiliations of the developers, sponsors, and owners [6] (if authors/evaluators are owners or developer of the software, this needs to be declared in a “Conflict of interest” section or mentioned elsewhere in the manuscript).

	1	2	3	4	5	
subitem not at all important	○	○	○	◉	○	essential

Does your paper address subitem 5-i?

Copy and paste relevant sections from the manuscript (include quotes in quotation marks "like this" to indicate direct quotes from your manuscript), or elaborate on this item by providing additional information not in the ms, or briefly explain why the item is not applicable/relevant for your study

"Message content was developed by the study team (experts on AYA medication adherence, behavior change theory, and pediatric nephrology)..." "Text messages containing the Reminder+COM-B message intervention or Reminder-only active control messages were sent from a single study number via REDCap’s Twilio interface."

5-ii) Describe the history/development process

Describe the history/development process of the application and previous formative evaluations (e.g., focus groups, usability testing), as these will have an impact on adoption/use rates and help with interpreting results.

	1	2	3	4	5	
subitem not at all important	○	○	○	●	○	essential

Does your paper address subitem 5-ii?

Copy and paste relevant sections from the manuscript (include quotes in quotation marks "like this" to indicate direct quotes from your manuscript), or elaborate on this item by providing additional information not in the ms, or briefly explain why the item is not applicable/relevant for your study

See "Phase 1: Reminder+COM-B Message Intervention Development" section for these details.

5-iii) Revisions and updating

Revisions and updating. Clearly mention the date and/or version number of the application/intervention (and comparator, if applicable) evaluated, or describe whether the intervention underwent major changes during the evaluation process, or whether the development and/or content was "frozen" during the trial. Describe dynamic components such as news feeds or changing content which may have an impact on the replicability of the intervention (for unexpected events see item 3b).

	1	2	3	4	5	
subitem not at all important	○	○	●	○	○	essential

Does your paper address subitem 5-iii?

Copy and paste relevant sections from the manuscript (include quotes in quotation marks "like this" to indicate direct quotes from your manuscript), or elaborate on this item by providing additional information not in the ms, or briefly explain why the item is not applicable/relevant for your study

"Messages rated by <85% of AYAs as acceptable, effective, helpful, or understood were excluded or revised before inclusion in the final pool tested in Phase 2. The final Reminder+COM-B message pool included 14 messages targeting adherence capability (e.g., “Tip: Put your medicine in a safe place you look each day (like the kitchen counter or by your bed) to help remember to take them”), 14 messages targeting adherence motivation (e.g., “Think about your future goals and how being healthier by taking your medicine may help you achieve them!”), and a simple reminder targeting adherence opportunity (“Please remember to take your medicine”)."

5-iv) Quality assurance methods

Provide information on quality assurance methods to ensure accuracy and quality of information provided [1], if applicable.

	1	2	3	4	5	
subitem not at all important	○	○	◉	○	○	essential

Does your paper address subitem 5-iv?

Copy and paste relevant sections from the manuscript (include quotes in quotation marks "like this" to indicate direct quotes from your manuscript), or elaborate on this item by providing additional information not in the ms, or briefly explain why the item is not applicable/relevant for your study

"All AYAs in the final sample completed Phase 2 pilot RCT procedures and all AYAs in the Reminder+COM-B message intervention group completed a Phase 3 qualitative interview. All AYAs used the electronic pill bottles without technical or reported problems. No AYAs in either group asked for the messages to stop or reported that the messages bothered them. No AYAs in the Reminder+COM-B message intervention group reported that the messages reduced their desire to take their medicine."

5-v) Ensure replicability by publishing the source code, and/or providing screenshots/screen-capture video, and/or providing flowcharts of the algorithms used

Ensure replicability by publishing the source code, and/or providing screenshots/screen-capture video, and/or providing flowcharts of the algorithms used. Replicability (i.e., other researchers should in principle be able to replicate the study) is a hallmark of scientific reporting.

	1	2	3	4	5	
subitem not at all important	○	○	◉	○	○	essential

Does your paper address subitem 5-v?

Copy and paste relevant sections from the manuscript (include quotes in quotation marks "like this" to indicate direct quotes from your manuscript), or elaborate on this item by providing additional information not in the ms, or briefly explain why the item is not applicable/relevant for your study

See Phases 1-3 in Method section. These sections clearly outline the steps taken in each phase of the study and describe how the intervention and active control messages were delivered for replicability.

5-vi) Digital preservation

Digital preservation: Provide the URL of the application, but as the intervention is likely to change or disappear over the course of the years; also make sure the intervention is archived (Internet Archive, webcitation.org, and/or publishing the source code or screenshots/videos alongside the article). As pages behind login screens cannot be archived, consider creating demo pages which are accessible without login.

	1	2	3	4	5	
subitem not at all important	○	○	◉	○	○	essential

Does your paper address subitem 5-vi?

Copy and paste relevant sections from the manuscript (include quotes in quotation marks "like this" to indicate direct quotes from your manuscript), or elaborate on this item by providing additional information not in the ms, or briefly explain why the item is not applicable/relevant for your study

N/A: Our intervention relied on SMS text messages, thus there is no URL to link to an app.

5-vii) Access

Access: Describe how participants accessed the application, in what setting/context, if they had to pay (or were paid) or not, whether they had to be a member of specific group. If known, describe how participants obtained “access to the platform and Internet” [1]. To ensure access for editors/reviewers/readers, consider to provide a “backdoor” login account or demo mode for reviewers/readers to explore the application (also important for archiving purposes, see vi).

	1	2	3	4	5	
subitem not at all important	○	○	●	○	○	essential

Does your paper address subitem 5-vii? *

Copy and paste relevant sections from the manuscript (include quotes in quotation marks "like this" to indicate direct quotes from your manuscript), or elaborate on this item by providing additional information not in the ms, or briefly explain why the item is not applicable/relevant for your study

"Text messages containing the Reminder+COM-B message intervention or Reminder-only active control messages were sent from a single study number via REDCap’s Twilio interface." No app was used in this study.

5-viii) Mode of delivery, features/functionalities/components of the intervention and comparator, and the theoretical framework

Describe mode of delivery, features/functionalities/components of the intervention and comparator, and the theoretical framework [6] used to design them (instructional strategy [1], behaviour change techniques, persuasive features, etc., see e.g., [7, 8] for terminology). This includes an in-depth description of the content (including where it is coming from and who developed it) [1],” whether [and how] it is tailored to individual circumstances and allows users to track their progress and receive feedback” [6]. This also includes a description of communication delivery channels and – if computer-mediated communication is a component – whether communication was synchronous or asynchronous [6]. It also includes information on presentation strategies [1], including page design principles, average amount of text on pages, presence of hyperlinks to other resources, etc. [1].

	1	2	3	4	5	
subitem not at all important	○	○	○	●	○	essential

Does your paper address subitem 5-viii? *

Copy and paste relevant sections from the manuscript (include quotes in quotation marks "like this" to indicate direct quotes from your manuscript), or elaborate on this item by providing additional information not in the ms, or briefly explain why the item is not applicable/relevant for your study

"Phase 1: Reminder+COM-B Message Intervention Development. Message content was developed by the study team (experts on AYA medication adherence, behavior change theory, and pediatric nephrology) to target COM-B model components [19] and incorporate effective public health communication strategies (e.g., gain versus loss framing, minimizing use of fear appeals) [21-24]. Messages were written at ≤5th grade reading level and contained <140 characters. Messages targeting the COM-B model's capability and motivation components were based on validated self-report measures of AYA adherence barriers and beliefs [25,26]. The COM-B model's opportunity component was targeted with a simple medicine reminder message. Pediatric nephrologists (N=6) at the study site provided feedback on messages' medical accuracy.
The initial intervention message pool was presented to 10 AYAs with CKD (M age=16.50 years, SD=3.41, range=12-21 years) during a semi-structured telephone interview (~60 minutes; audio-recorded for this study). AYAs rated messages on acceptability, effectiveness, helpfulness, and comprehension and provided open-ended suggestions for improving content. AYAs received $20 each for completing the interviews.
Messages rated by <85% of AYAs as acceptable, effective, helpful, or understood were excluded or revised before inclusion in the final pool tested in Phase 2. The final Reminder+COM-B message pool included 14 messages targeting adherence capability (e.g., "Tip: Put your medicine in a safe place you look each day (like the kitchen counter or by your bed) to help remember to take them"), 14 messages targeting adherence motivation (e.g., "Think about your future goals and how being healthier by taking your medicine may help you achieve them!"), and a simple reminder targeting adherence opportunity ("Please remember to take your medicine"). The Reminder+COM-B message intervention involved sending AYAs a daily message bundle, which included the opportunity message (simple reminder) and a capability or motivation message (alternated each day) at the time(s) when the AYA reportedly took their antihypertensive medicine.
Reminder-only Message Active Control Condition. The Reminder-only message active control condition involved sending AYAs the daily opportunity message only (simple reminder). Reminder messages were sent at the time(s) when each AYA reportedly took their antihypertensive medicine."
"AYAs were informed that the study text messages would be sent to their mobile phone at some point during the pilot RCT but were not provided with a specific date. Text messages containing the Reminder+COM-B message intervention or Reminder-only active control messages were sent from a single study number via REDCap's Twilio interface."

5-ix) Describe use parameters

Describe use parameters (e.g., intended “doses” and optimal timing for use). Clarify what instructions or recommendations were given to the user, e.g., regarding timing, frequency, heaviness of use, if any, or was the intervention used ad libitum.

	1	2	3	4	5	
subitem not at all important	○	○	◉	○	○	essential

Does your paper address subitem 5-ix?

Copy and paste relevant sections from the manuscript (include quotes in quotation marks "like this" to indicate direct quotes from your manuscript), or elaborate on this item by providing additional information not in the ms, or briefly explain why the item is not applicable/relevant for your study

"The Reminder+COM-B message intervention involved sending AYAs a daily message bundle, which included the opportunity message (simple reminder) and a capability or motivation message (alternated each day) at the time(s) when the AYA reportedly took their antihypertensive medicine...The Reminder-only message active control condition involved sending AYAs the daily opportunity message only (simple reminder). Reminder messages were sent at the time(s) when each AYA reportedly took their antihypertensive medicine."

5-x) Clarify the level of human involvement

Clarify the level of human involvement (care providers or health professionals, also technical assistance) in the e-intervention or as co-intervention (detail number and expertise of professionals involved, if any, as well as “type of assistance offered, the timing and frequency of the support, how it is initiated, and the medium by which the assistance is delivered”. It may be necessary to distinguish between the level of human involvement required for the trial, and the level of human involvement required for a routine application outside of a RCT setting (discuss under item 21 – generalizability).

	1	2	3	4	5	
subitem not at all important	○	○	○	○	◉	essential

Does your paper address subitem 5-x?

Copy and paste relevant sections from the manuscript (include quotes in quotation marks "like this" to indicate direct quotes from your manuscript), or elaborate on this item by providing additional information not in the ms, or briefly explain why the item is not applicable/relevant for your study

"...the messages were delivered remotely via text message rather than face-to-face."

5-xi) Report any prompts/reminders used

Report any prompts/reminders used: Clarify if there were prompts (letters, emails, phone calls, SMS) to use the application, what triggered them, frequency etc. It may be necessary to distinguish between the level of prompts/reminders required for the trial, and the level of prompts/reminders for a routine application outside of a RCT setting (discuss under item 21 – generalizability).

	1	2	3	4	5	
subitem not at all important	○	○	○	○	◉	essential

Does your paper address subitem 5-xi? *

Copy and paste relevant sections from the manuscript (include quotes in quotation marks "like this" to indicate direct quotes from your manuscript), or elaborate on this item by providing additional information not in the ms, or briefly explain why the item is not applicable/relevant for your study

N/A: Our intervention involved sending daily SMS text messages directly to the participant on a set schedule. There was no app that they were asked to engage with.

5-xii) Describe any co-interventions (incl. training/support)

Describe any co-interventions (incl. training/support): Clearly state any interventions that are provided in addition to the targeted eHealth intervention, as ehealth intervention may not be designed as stand-alone intervention. This includes training sessions and support [1]. It may be necessary to distinguish between the level of training required for the trial, and the level of training for a routine application outside of a RCT setting (discuss under item 21 – generalizability.

	1	2	3	4	5	
subitem not at all important	○	○	○	◉	○	essential

Does your paper address subitem 5-xii? *

Copy and paste relevant sections from the manuscript (include quotes in quotation marks "like this" to indicate direct quotes from your manuscript), or elaborate on this item by providing additional information not in the ms, or briefly explain why the item is not applicable/relevant for your study

N/A: Our study did not involve any co-interventions. We clearly describe the Reminder+COM-B message intervention and the Reminder-only Active Control condition that were both tested in this study (see Methods section)

6a) Completely defined pre-specified primary and secondary outcome measures, including how and when they were assessed

Does your paper address CONSORT subitem 6a? *

Copy and paste relevant sections from the manuscript (include quotes in quotation marks "like this" to indicate direct quotes from your manuscript), or elaborate on this item by providing additional information not in the ms, or briefly explain why the item is not applicable/relevant for your study

"Objective Medication Adherence. AdhereTech bottles electronically-assessed daily antihypertensive medication adherence via a cellular-connected cap that recorded the date and time when the bottle cap was opened and closed. Timestamps were automatically transferred to AdhereTech's secure online portal. If more than one antihypertensive medication was prescribed, AYAs selected which medicine they wanted to monitor in the AdhereTech bottle. Daily adherence was coded as whether the bottle was opened that day (1) or not (0). If the AYA took the monitored medicine twice-a-day, daily adherence was coded as whether the bottle was opened twice that day (1) or not (0). Average adherence was calculated as the number of bottle cap openings recorded divided by the total number of expected openings based on the prescribed regimen and days in the monitoring period.

Surveys of Adherence Capability and Motivation

Adherence Capability. To represent capability as defined in the COM-B model (e.g., skills, knowledge, ability to take medicine) [19], the Riekert Self-Efficacy Scale [20] and the Adolescent Medication Barriers Scale (AMBS) [25] were administered.

The Riekert Self-Efficacy Scale (12 items) asked AYAs to rate their ability to take medicine in different situations on a 10-point Likert scale ranging from Not at all sure to Completely sure (e.g., "How sure are you that you can take your blood pressure medicine the way your doctor said when you want to do something else?"). Ratings were summed and divided by 12 for a scaled score ranging from 1 to 10 (higher scores=higher self-efficacy). Internal consistency in this study ranged from .94-.95.

The AMBS (17 items) assessed AYAs' adherence barriers. AYAs rated each item using a 5-point Likert scale ranging from Strongly disagree to Strongly agree (e.g., "I find it hard to stick to a fixed medication schedule"). The AMBS contains subscales but only the Total score was analyzed (ratings were summed and divided by 17 for a scaled score ranging from 1 to 5; higher scores=higher adherence barriers). Internal consistency in this study ranged from .81-.89.

Adherence Motivation. To represent motivation as defined in the COM-B model (conscious, reflective, and learned reasons for taking medicine as prescribed) [19], several scales from the Beliefs About Medication Scale [26-28] were administered.

The Positive Outcome Expectancies (POE; 20 items) and Negative Outcome Expectancies (NOE; 13 items) scales assessed AYAs' expectations of favorable/unfavorable outcomes for taking medications as prescribed (e.g., "When I take my medicine the way I should, I feel well enough to do things I enjoy" [POE]; "Taking my medicine the way I should makes me miss out on doing fun things" [NOE]). Items are rated on a 7-point Likert Scale ranging from Definitely do not agree to Definitely agree. Item ratings were summed and divided by the number of scale items to obtain scaled scores ranging from 1 to 7 (higher scores=more positive expectations [POE] or more negative expectations [NOE]). Internal consistency in this study ranged from .85-.87 for the POE and .90-.92 for the NOE.

The Adherence Motivation and Importance scales (3 items each) assessed AYAs' perspectives on the importance of taking medicine (e.g., "How important do you think it is for you to take your blood pressure medication the way the doctor said when you feel just fine?") and motivation to do so (e.g., "How much do you want to take your blood pressure medication the way the doctor said everyday?") on a 10-point Likert scale. Item ratings were

medication the way the doctor said everyday?") on a 10-point Likert scale. Item ratings were summed and divided by 3 to obtain scaled scores (higher scores=higher importance or motivation). Internal consistency in this study ranged from .76-.96 for Importance and .84-.96 for Motivation.

Phase 3: Qualitative Interviews. Following completion of Phase 2, AYAs in the Reminder+COM-B message intervention group were invited to complete audio-recorded and transcribed qualitative interviews (~30 minutes) by telephone. The interviewer (C.K.E.) followed an iterative interview guide evaluating AYAs' perceptions of the intervention, including mechanisms of behavior change and suggestions for improving the intervention."

6a-i) Online questionnaires: describe if they were validated for online use and apply CHERRIES items to describe how the questionnaires were designed/deployed

If outcomes were obtained through online questionnaires, describe if they were validated for online use and apply CHERRIES items to describe how the questionnaires were designed/deployed [9].

	1	2	3	4	5	
subitem not at all important	○	○	●	○	○	essential

Does your paper address subitem 6a-i?

Copy and paste relevant sections from manuscript text

"A brief demographic survey was sent to AYAs ≥18 years old and caregivers of AYAs <18 years old via REDCap. AYAs completed online surveys (Qualtrics) at baseline and after the pilot RCT ended."

6a-ii) Describe whether and how “use” (including intensity of use/dosage) was defined/measured/monitored

Describe whether and how “use” (including intensity of use/dosage) was defined/measured/monitored (logins, logfile analysis, etc.). Use/adoption metrics are important process outcomes that should be reported in any ehealth trial.

	1	2	3	4	5	
subitem not at all important	○	○	○	◉	○	essential

Does your paper address subitem 6a-ii?

Copy and paste relevant sections from manuscript text

"All AYAs in the final sample completed Phase 2 pilot RCT procedures and all AYAs in the Reminder+COM-B message intervention group completed a Phase 3 qualitative interview. All AYAs used the electronic pill bottles without technical or reported problems. No AYAs in either group asked for the messages to stop or reported that the messages bothered them. No AYAs in the Reminder+COM-B message intervention group reported that the messages reduced their desire to take their medicine."

6a-iii) Describe whether, how, and when qualitative feedback from participants was obtained

Describe whether, how, and when qualitative feedback from participants was obtained (e.g., through emails, feedback forms, interviews, focus groups).

	1	2	3	4	5	
subitem not at all important	○	○	○	○	◉	essential

Does your paper address subitem 6a-iii?

Copy and paste relevant sections from manuscript text

"Phase 3: Qualitative Interviews. Following completion of Phase 2, AYAs in the Reminder+COM-B message intervention group were invited to complete audio-recorded and transcribed qualitative interviews (~30 minutes) by telephone. The interviewer (C.K.E.) followed an iterative interview guide evaluating AYAs' perceptions of the intervention, including mechanisms of behavior change and suggestions for improving the intervention. Interviews lasted, on average, 31 minutes (SD=11). No repeat interviews were conducted. AYAs received $20 for their time."

6b) Any changes to trial outcomes after the trial commenced, with reasons

Does your paper address CONSORT subitem 6b? *

Copy and paste relevant sections from the manuscript (include quotes in quotation marks "like this" to indicate direct quotes from your manuscript), or elaborate on this item by providing additional information not in the ms, or briefly explain why the item is not applicable/relevant for your study

N/A: no changes were made to our trial outcome after the trial commenced.

7a) How sample size was determined

NPT: When applicable, details of whether and how the clustering by care provides or centers was addressed

7a-i) Describe whether and how expected attrition was taken into account when calculating the sample size

Describe whether and how expected attrition was taken into account when calculating the sample size.

	1	2	3	4	5	
subitem not at all important	○	○	●	○	○	essential

Does your paper address subitem 7a-i?

Copy and paste relevant sections from manuscript title (include quotes in quotation marks "like this" to indicate direct quotes from your manuscript), or elaborate on this item by providing additional information not in the ms, or briefly explain why the item is not applicable/relevant for your study

Our study is a single-recruitment site pilot investigation and therefore, the sample size is smaller. We clearly report our screening, recruitment, and enrollment data in Figure 1, which reports attrition rates.

7b) When applicable, explanation of any interim analyses and stopping guidelines

Does your paper address CONSORT subitem 7b? *

Copy and paste relevant sections from the manuscript (include quotes in quotation marks "like this" to indicate direct quotes from your manuscript), or elaborate on this item by providing additional information not in the ms, or briefly explain why the item is not applicable/relevant for your study

N/A: Our study did not involve interim analyses or stopping guidelines.

8a) Method used to generate the random allocation sequence

NPT: When applicable, how care providers were allocated to each trial group

Does your paper address CONSORT subitem 8a? *

Copy and paste relevant sections from the manuscript (include quotes in quotation marks "like this" to indicate direct quotes from your manuscript), or elaborate on this item by providing additional information not in the ms, or briefly explain why the item is not applicable/relevant for your study

"AYAs were randomized to the Reminder+COM-B message intervention or Reminder-only message active control group and received their respective group's messages for the last 4 weeks of the pilot RCT. AYAs were randomized in a 1:1 basis to either group. A random number generator was used for simple randomization and AYAs were assigned consecutively to a group. Blinding the study team to group assignment was not possible; however, the messages were delivered remotely via text message rather than face-to-face."

8b) Type of randomisation; details of any restriction (such as blocking and block size)

Does your paper address CONSORT subitem 8b? *

Copy and paste relevant sections from the manuscript (include quotes in quotation marks "like this" to indicate direct quotes from your manuscript), or elaborate on this item by providing additional information not in the ms, or briefly explain why the item is not applicable/relevant for your study

"AYAs were randomized in a 1:1 basis to either group. A random number generator was used for simple randomization and AYAs were assigned consecutively to a group."

9) Mechanism used to implement the random allocation sequence (such as sequentially numbered containers), describing any steps taken to conceal the sequence until interventions were assigned

Does your paper address CONSORT subitem 9? *

Copy and paste relevant sections from the manuscript (include quotes in quotation marks "like this" to indicate direct quotes from your manuscript), or elaborate on this item by providing additional information not in the ms, or briefly explain why the item is not applicable/relevant for your study

"AYAs were randomized to the Reminder+COM-B message intervention or Reminder-only message active control group and received their respective group’s messages for the last 4 weeks of the pilot RCT. AYAs were randomized in a 1:1 basis to either group. A random number generator was used for simple randomization and AYAs were assigned consecutively to a group. Blinding the study team to group assignment was not possible; however, the messages were delivered remotely via text message rather than face-to-face."

10) Who generated the random allocation sequence, who enrolled participants, and who assigned participants to interventions

Does your paper address CONSORT subitem 10? *

Copy and paste relevant sections from the manuscript (include quotes in quotation marks "like this" to indicate direct quotes from your manuscript), or elaborate on this item by providing additional information not in the ms, or briefly explain why the item is not applicable/relevant for your study

"AYAs were randomized to the Reminder+COM-B message intervention or Reminder-only message active control group and received their respective group's messages for the last 4 weeks of the pilot RCT. AYAs were randomized in a 1:1 basis to either group. A random number generator was used for simple randomization and AYAs were assigned consecutively to a group. Blinding the study team to group assignment was not possible; however, the messages were delivered remotely via text message rather than face-to-face."

11a) If done, who was blinded after assignment to interventions (for example, participants, care providers, those assessing outcomes) and how

NPT: Whether or not administering co-interventions were blinded to group assignment

11a-i) Specify who was blinded, and who wasn't

Specify who was blinded, and who wasn't. Usually, in web-based trials it is not possible to blind the participants [1, 3] (this should be clearly acknowledged), but it may be possible to blind outcome assessors, those doing data analysis or those administering co-interventions (if any).

	1	2	3	4	5	
subitem not at all important	○	○	○	○	◉	essential

Does your paper address subitem 11a-i? *

Copy and paste relevant sections from the manuscript (include quotes in quotation marks "like this" to indicate direct quotes from your manuscript), or elaborate on this item by providing additional information not in the ms, or briefly explain why the item is not applicable/relevant for your study

"Blinding the study team to group assignment was not possible; however, the messages were delivered remotely via text message rather than face-to-face."

11a-ii) Discuss e.g., whether participants knew which intervention was the "intervention of interest" and which one was the "comparator"

Informed consent procedures (4a-ii) can create biases and certain expectations - discuss e.g., whether participants knew which intervention was the "intervention of interest" and which one was the "comparator".

	1	2	3	4	5	
subitem not at all important	○	○	○	◉	○	essential

Does your paper address subitem 11a-ii?

Copy and paste relevant sections from the manuscript (include quotes in quotation marks "like this" to indicate direct quotes from your manuscript), or elaborate on this item by providing additional information not in the ms, or briefly explain why the item is not applicable/relevant for your study

"At the end of the 8-weeks, AYAs in the Reminder+COM-B message intervention group completed a qualitative interview (see Qualitative Interviews section)."

11b) If relevant, description of the similarity of interventions

(this item is usually not relevant for ehealth trials as it refers to similarity of a placebo or sham intervention to a active medication/intervention)

Does your paper address CONSORT subitem 11b? *

Copy and paste relevant sections from the manuscript (include quotes in quotation marks "like this" to indicate direct quotes from your manuscript), or elaborate on this item by providing additional information not in the ms, or briefly explain why the item is not applicable/relevant for your study

"The Reminder+COM-B message intervention involved sending AYAs a daily message bundle, which included the opportunity message (simple reminder) and a capability or motivation message (alternated each day) at the time(s) when the AYA reportedly took their antihypertensive medicine.
Reminder-only Message Active Control Condition. The Reminder-only message active control condition involved sending AYAs the daily opportunity message only (simple reminder). Reminder messages were sent at the time(s) when each AYA reportedly took their antihypertensive medicine."

12a) Statistical methods used to compare groups for primary and secondary outcomes

NPT: When applicable, details of whether and how the clustering by care providers or centers was addressed

Does your paper address CONSORT subitem 12a? *

Copy and paste relevant sections from the manuscript (include quotes in quotation marks "like this" to indicate direct quotes from your manuscript), or elaborate on this item by providing additional information not in the ms, or briefly explain why the item is not applicable/relevant for your study

"Descriptive statistics (mean, standard deviation, range) were calculated for primary study variables by randomization group and measurement time point. For hypothesis testing, statistical significance was assumed when P<.05. A linear mixed model (PROC MIXED; SAS 9.4 Software, Cary, NC) was used to examine whether changes in daily adherence over time differed by group allocation, controlling for AYA age, gender, race, and hypertension diagnosis (covariates were based on variables commonly associated with pediatric adherence [4] including AYAs with CKD [20]). Change in daily adherence during both baseline and post-randomization phases were initially examined to determine the functional form of the time variable (linear or quadratic) and whether to include an individual-level random slope. The best fitting model for time was selected using the Akaike Information Criterion (AIC) values [29]. Based on this initial examination, time was modeled as a linear function with an individual-level random slope during baseline (AIC=750.6) and post-randomization (AIC=750.9). Models were fitted using restricted maximum likelihood estimation. An autoregressive covariance structure was used to account for expected correlations between repeated daily adherence assessments within participants. There were no missing adherence data.
Repeated-measures ANOVA (IBM SPSS Statistics Version 26) with one within-subject factor (time point) and one between-subject factor (treatment group) was used to examine whether change in mean adherence during baseline and post-randomization and pre-post mean survey scores differed by group (controlled for AYA age, gender, race, and hypertension diagnosis [4,20]). The magnitude of within-treatment group change between study time points was examined with Cohen’s d."

12a-i) Imputation techniques to deal with attrition / missing values

Imputation techniques to deal with attrition / missing values: Not all participants will use the intervention/comparator as intended and attrition is typically high in ehealth trials. Specify how participants who did not use the application or dropped out from the trial were treated in the statistical analysis (a complete case analysis is strongly discouraged, and simple imputation techniques such as LOCF may also be problematic [4]).

	1	2	3	4	5	
subitem not at all important	○	○	○	○	◉	essential

Does your paper address subitem 12a-i? *

Copy and paste relevant sections from the manuscript (include quotes in quotation marks "like this" to indicate direct quotes from your manuscript), or elaborate on this item by providing additional information not in the ms, or briefly explain why the item is not applicable/relevant for your study

"There were no missing adherence data."

12b) Methods for additional analyses, such as subgroup analyses and adjusted analyses

Does your paper address CONSORT subitem 12b? *

Copy and paste relevant sections from the manuscript (include quotes in quotation marks "like this" to indicate direct quotes from your manuscript), or elaborate on this item by providing additional information not in the ms, or briefly explain why the item is not applicable/relevant for your study

"The magnitude of within-treatment group change between study time points was examined with Cohen’s d."

X26) REB/IRB Approval and Ethical Considerations [recommended as subheading under "Methods"] (not a CONSORT item)

X26-i) Comment on ethics committee approval

	1	2	3	4	5	
subitem not at all important	○	○	○	○	◉	essential

Does your paper address subitem X26-i?

Copy and paste relevant sections from the manuscript (include quotes in quotation marks "like this" to indicate direct quotes from your manuscript), or elaborate on this item by providing additional information not in the ms, or briefly explain why the item is not applicable/relevant for your study

The Johns Hopkins School of Medicine Institutional Review Board approved all study procedures prior to recruitment, which occurred at a single pediatric nephrology clinic (October 2018-November 2019).

x26-ii) Outline informed consent procedures

Outline informed consent procedures e.g., if consent was obtained offline or online (how? Checkbox, etc.?), and what information was provided (see 4a-ii). See [6] for some items to be included in informed consent documents.

	1	2	3	4	5	
subitem not at all important	○	○	○	○	◉	essential

Does your paper address subitem X26-ii?

Copy and paste relevant sections from the manuscript (include quotes in quotation marks "like this" to indicate direct quotes from your manuscript), or elaborate on this item by providing additional information not in the ms, or briefly explain why the item is not applicable/relevant for your study

Informed consent/assent procedures were conducted with participants during a telephone call with study staff (AYAs >18 years old provided consent for themselves; for AYAs <18 years old, a primary caregiver provided informed consent for their child and the AYA provided informed assent for themselves).

X26-iii) Safety and security procedures

Safety and security procedures, incl. privacy considerations, and any steps taken to reduce the likelihood or detection of harm (e.g., education and training, availability of a hotline)

	1	2	3	4	5	
subitem not at all important	○	○	○	◉	○	essential

Does your paper address subitem X26-iii?

Copy and paste relevant sections from the manuscript (include quotes in quotation marks "like this" to indicate direct quotes from your manuscript), or elaborate on this item by providing additional information not in the ms, or briefly explain why the item is not applicable/relevant for your study

N/A: Participants were informed about the limits of confidentiality due to the nature of being in a research study as well as the privacy measures taken by the study team to reduce this risk and protect data collected, and were provided with the study team's (including the PI) direct telephone number and email address to report any issues or concerns. There were no additional education or training or availability of a hotline offered for this purpose.

RESULTS

13a) For each group, the numbers of participants who were randomly assigned, received intended treatment, and were analysed for the primary outcome

NPT: The number of care providers or centers performing the intervention in each group and the number of patients treated by each care provider in each center

Does your paper address CONSORT subitem 13a? *

Copy and paste relevant sections from the manuscript (include quotes in quotation marks "like this" to indicate direct quotes from your manuscript), or elaborate on this item by providing additional information not in the ms, or briefly explain why the item is not applicable/relevant for your study

"Hence, 34 AYAs (M age = 16.59 years, SD = 3.26, range =11-21 years) completed Phase 2 (Reminder+COM-B message intervention group=18, Reminder-only message active control group=16)."

13b) For each group, losses and exclusions after randomisation, together with reasons

Does your paper address CONSORT subitem 13b? (NOTE: Preferably, this is shown in a CONSORT flow diagram) *

Copy and paste relevant sections from the manuscript (include quotes in quotation marks "like this" to indicate direct quotes from your manuscript), or elaborate on this item by providing additional information not in the ms, or briefly explain why the item is not applicable/relevant for your study

These data are reported in Figure 1 and the Results section: "All AYAs in the final sample completed Phase 2 pilot RCT procedures and all AYAs in the Reminder+COM-B message intervention group completed a Phase 3 qualitative interview."

13b-i) Attrition diagram

Strongly recommended: An attrition diagram (e.g., proportion of participants still logging in or using the intervention/comparator in each group plotted over time, similar to a survival curve) or other figures or tables demonstrating usage/dose/engagement.

	1	2	3	4	5	
subitem not at all important	○	○	○	○	◉	essential

Does your paper address subitem 13b-i?

Copy and paste relevant sections from the manuscript or cite the figure number if applicable (include quotes in quotation marks "like this" to indicate direct quotes from your manuscript), or elaborate on this item by providing additional information not in the ms, or briefly explain why the item is not applicable/relevant for your study

"All AYAs in the final sample completed Phase 2 pilot RCT procedures and all AYAs in the Reminder+COM-B message intervention group completed a Phase 3 qualitative interview. All AYAs used the electronic pill bottles without technical or reported problems. No AYAs in either group asked for the messages to stop or reported that the messages bothered them. No AYAs in the Reminder+COM-B message intervention group reported that the messages reduced their desire to take their medicine." These data are also reported in Figure 1.

14a) Dates defining the periods of recruitment and follow-up

Does your paper address CONSORT subitem 14a? *

Copy and paste relevant sections from the manuscript (include quotes in quotation marks "like this" to indicate direct quotes from your manuscript), or elaborate on this item by providing additional information not in the ms, or briefly explain why the item is not applicable/relevant for your study

"The Johns Hopkins School of Medicine Institutional Review Board approved all study procedures prior to recruitment, which occurred at a single pediatric nephrology clinic (October 2018-November 2019)." "The first 4 weeks of the pilot RCT (baseline) evaluated AYA adherence without sending any messages. AYAs were randomized to the Reminder+COM-B message intervention or Reminder-only message active control group and received their respective group's messages for the last 4 weeks of the pilot RCT." "At the end of the 8-weeks, AYAs in the Reminder+COM-B message intervention group completed a qualitative interview (see Qualitative Interviews section). AYAs in both groups received $40 each for completing the pilot RCT and pre- and post-surveys"

14a-i) Indicate if critical “secular events” fell into the study period

Indicate if critical “secular events” fell into the study period, e.g., significant changes in Internet resources available or “changes in computer hardware or Internet delivery resources”

	1	2	3	4	5	
subitem not at all important	○	○	○	○	○	essential

Does your paper address subitem 14a-i?

Copy and paste relevant sections from the manuscript (include quotes in quotation marks "like this" to indicate direct quotes from your manuscript), or elaborate on this item by providing additional information not in the ms, or briefly explain why the item is not applicable/relevant for your study

N/A

14b) Why the trial ended or was stopped (early)

Does your paper address CONSORT subitem 14b? *

Copy and paste relevant sections from the manuscript (include quotes in quotation marks "like this" to indicate direct quotes from your manuscript), or elaborate on this item by providing additional information not in the ms, or briefly explain why the item is not applicable/relevant for your study

N/A--the trial was completed as planned (no early stopping)

15) A table showing baseline demographic and clinical characteristics for each group

NPT: When applicable, a description of care providers (case volume, qualification, expertise, etc.) and centers (volume) in each group

Does your paper address CONSORT subitem 15? *

Copy and paste relevant sections from the manuscript (include quotes in quotation marks "like this" to indicate direct quotes from your manuscript), or elaborate on this item by providing additional information not in the ms, or briefly explain why the item is not applicable/relevant for your study

See Table 1

15-i) Report demographics associated with digital divide issues

In ehealth trials it is particularly important to report demographics associated with digital divide issues, such as age, education, gender, social-economic status, computer/Internet/ehealth literacy of the participants, if known.

	1	2	3	4	5	
subitem not at all important	○	○	○	○	◉	essential

Does your paper address subitem 15-i? *

Copy and paste relevant sections from the manuscript (include quotes in quotation marks "like this" to indicate direct quotes from your manuscript), or elaborate on this item by providing additional information not in the ms, or briefly explain why the item is not applicable/relevant for your study

See Table 1--we report all available demographic data for participants here.

16) For each group, number of participants (denominator) included in each analysis and whether the analysis was by original assigned groups

16-i) Report multiple “denominators” and provide definitions

Report multiple “denominators” and provide definitions: Report N’s (and effect sizes) “across a range of study participation [and use] thresholds” [1], e.g., N exposed, N consented, N used more than x times, N used more than y weeks, N participants “used” the intervention/comparator at specific pre-defined time points of interest (in absolute and relative numbers per group). Always clearly define “use” of the intervention.

	1	2	3	4	5	
subitem not at all important	○	○	○	○	◉	essential

Does your paper address subitem 16-i? *

Copy and paste relevant sections from the manuscript (include quotes in quotation marks "like this" to indicate direct quotes from your manuscript), or elaborate on this item by providing additional information not in the ms, or briefly explain why the item is not applicable/relevant for your study

See Table 1 for Ns at each stage of the study and denominators for final analyses.

16-ii) Primary analysis should be intent-to-treat

Primary analysis should be intent-to-treat, secondary analyses could include comparing only “users”, with the appropriate caveats that this is no longer a randomized sample (see 18-i).

	1	2	3	4	5	
subitem not at all important	○	○	○	○	◉	essential

Does your paper address subitem 16-ii?

Copy and paste relevant sections from the manuscript (include quotes in quotation marks "like this" to indicate direct quotes from your manuscript), or elaborate on this item by providing additional information not in the ms, or briefly explain why the item is not applicable/relevant for your study

"34 AYAs (M age = 16.59 years, SD = 3.26, range =11-21 years) completed Phase 2 (Reminder+COM-B message intervention group=18, Reminder-only message active control group=16)." These 34 AYAs were all included for final analyses.

17a) For each primary and secondary outcome, results for each group, and the estimated effect size and its precision (such as 95% confidence interval)

Does your paper address CONSORT subitem 17a? *

Copy and paste relevant sections from the manuscript (include quotes in quotation marks "like this" to indicate direct quotes from your manuscript), or elaborate on this item by providing additional information not in the ms, or briefly explain why the item is not applicable/relevant for your study

See Table 2 for primary outcome results including 95% CIs.

17a-i) Presentation of process outcomes such as metrics of use and intensity of use

In addition to primary/secondary (clinical) outcomes, the presentation of process outcomes such as metrics of use and intensity of use (dose, exposure) and their operational definitions is critical. This does not only refer to metrics of attrition (13-b) (often a binary variable), but also to more continuous exposure metrics such as "average session length". These must be accompanied by a technical description how a metric like a "session" is defined (e.g., timeout after idle time) [1] (report under item 6a).

	1	2	3	4	5	
subitem not at all important	○	○	○	◉	○	essential

Does your paper address subitem 17a-i?

Copy and paste relevant sections from the manuscript (include quotes in quotation marks "like this" to indicate direct quotes from your manuscript), or elaborate on this item by providing additional information not in the ms, or briefly explain why the item is not applicable/relevant for your study

"All AYAs in the final sample completed Phase 2 pilot RCT procedures and all AYAs in the Reminder+COM-B message intervention group completed a Phase 3 qualitative interview. All AYAs used the electronic pill bottles without technical or reported problems. No AYAs in either group asked for the messages to stop or reported that the messages bothered them. No AYAs in the Reminder+COM-B message intervention group reported that the messages reduced their desire to take their medicine. The decline rate for study enrollment was relatively high (N=36) suggesting potential issues with acceptability in how the intervention and study procedures were presented during recruitment."

17b) For binary outcomes, presentation of both absolute and relative effect sizes is recommended

Does your paper address CONSORT subitem 17b? *

Copy and paste relevant sections from the manuscript (include quotes in quotation marks "like this" to indicate direct quotes from your manuscript), or elaborate on this item by providing additional information not in the ms, or briefly explain why the item is not applicable/relevant for your study

See Tables 2-3 for effect sizes of our outcomes.

18) Results of any other analyses performed, including subgroup analyses and adjusted analyses, distinguishing pre-specified from exploratory

Does your paper address CONSORT subitem 18? *

Copy and paste relevant sections from the manuscript (include quotes in quotation marks "like this" to indicate direct quotes from your manuscript), or elaborate on this item by providing additional information not in the ms, or briefly explain why the item is not applicable/relevant for your study

We reported post-hoc analyses for our adherence data to account for potential Hawthorne effects due to knowing one's adherence was being monitored.
"Post-hoc sensitivity analysis for baseline adherence. A post-hoc sensitivity analysis was conducted to exclude the first 7 days of adherence data due to potential Hawthorne effects of knowing one's adherence was electronically monitored. Results were similar whether the first 7 days of baseline data were excluded or included (adherence demonstrated a non-significant decline over time, no significant group by time interaction)."

"Post-hoc sensitivity analysis for mean baseline adherence. A post-hoc sensitivity analysis was run to exclude the first 7 days of adherence data from the baseline mean scores due to potential Hawthorne effects of knowing one's adherence was electronically monitored. Results were similar whether the first 7 days of baseline adherence data were excluded or included (no significant group by time interactions)."

18-i) Subgroup analysis of comparing only users

A subgroup analysis of comparing only users is not uncommon in ehealth trials, but if done, it must be stressed that this is a self-selected sample and no longer an unbiased sample from a randomized trial (see 16-iii).

	1	2	3	4	5	
subitem not at all important	○	○	●	○	○	essential

Does your paper address subitem 18-i?

Copy and paste relevant sections from the manuscript (include quotes in quotation marks "like this" to indicate direct quotes from your manuscript), or elaborate on this item by providing additional information not in the ms, or briefly explain why the item is not applicable/relevant for your study

N/A, we did not conduct these subgroup analyses of comparing only users.

19) All important harms or unintended effects in each group

(for specific guidance see CONSORT for harms)

Does your paper address CONSORT subitem 19? *

Copy and paste relevant sections from the manuscript (include quotes in quotation marks "like this" to indicate direct quotes from your manuscript), or elaborate on this item by providing additional information not in the ms, or briefly explain why the item is not applicable/relevant for your study

"All AYAs used the electronic pill bottles without technical or reported problems. No AYAs in either group asked for the messages to stop or reported that the messages bothered them. No AYAs in the Reminder+COM-B message intervention group reported that the messages reduced their desire to take their medicine."

19-i) Include privacy breaches, technical problems

Include privacy breaches, technical problems. This does not only include physical “harm” to participants, but also incidents such as perceived or real privacy breaches [1], technical problems, and other unexpected/unintended incidents. “Unintended effects” also includes unintended positive effects [2].

	1	2	3	4	5	
subitem not at all important	○	○	○	○	◉	essential

Does your paper address subitem 19-i?

Copy and paste relevant sections from the manuscript (include quotes in quotation marks "like this" to indicate direct quotes from your manuscript), or elaborate on this item by providing additional information not in the ms, or briefly explain why the item is not applicable/relevant for your study

"All AYAs used the electronic pill bottles without technical or reported problems. No AYAs in either group asked for the messages to stop or reported that the messages bothered them. No AYAs in the Reminder+COM-B message intervention group reported that the messages reduced their desire to take their medicine."

19-ii) Include qualitative feedback from participants or observations from staff/researchers

Include qualitative feedback from participants or observations from staff/researchers, if available, on strengths and shortcomings of the application, especially if they point to unintended/unexpected effects or uses. This includes (if available) reasons for why people did or did not use the application as intended by the developers.

	1	2	3	4	5	
subitem not at all important	○	○	○	○	◉	essential

Does your paper address subitem 19-ii?

Copy and paste relevant sections from the manuscript (include quotes in quotation marks "like this" to indicate direct quotes from your manuscript), or elaborate on this item by providing additional information not in the ms, or briefly explain why the item is not applicable/relevant for your study

See "Qualitative Interviews with AYAs in the Reminder+COM-B Message Intervention Group" section and Table 4.

DISCUSSION

22) Interpretation consistent with results, balancing benefits and harms, and considering other relevant evidence

NPT: In addition, take into account the choice of the comparator, lack of or partial blinding, and unequal expertise of care providers or centers in each group

22-i) Restate study questions and summarize the answers suggested by the data, starting with primary outcomes and process outcomes (use)

Restate study questions and summarize the answers suggested by the data, starting with primary outcomes and process outcomes (use).

	1	2	3	4	5	
subitem not at all important	○	○	○	○	◉	essential

Does your paper address subitem 22-i? *

Copy and paste relevant sections from the manuscript (include quotes in quotation marks "like this" to indicate direct quotes from your manuscript), or elaborate on this item by providing additional information not in the ms, or briefly explain why the item is not applicable/relevant for your study

"The current study investigated the preliminary efficacy of a mHealth messaging intervention based on the COM-B model [19] that aimed to promote objectively-measured antihypertensive medication adherence in AYAs with CKD and obtained qualitative stakeholder feedback on user experiences. Data quality were excellent, the Reminder+COM-B message intervention was feasible, and AYAs did not perceive the Reminder+COM-B messages to negatively impact their desire to take medicine. Our results suggest this intervention had stabilizing effects on daily adherence compared to a Reminder-only message active control group. Qualitative interviews provided insight into the Reminder+COM-B message intervention's mechanisms of behavior change and avenues for improving its efficacy."

22-ii) Highlight unanswered new questions, suggest future research

Highlight unanswered new questions, suggest future research.

	1	2	3	4	5	
subitem not at all important	○	○	○	○	◉	essential

Does your paper address subitem 22-ii?

Copy and paste relevant sections from the manuscript (include quotes in quotation marks "like this" to indicate direct quotes from your manuscript), or elaborate on this item by providing additional information not in the ms, or briefly explain why the item is not applicable/relevant for your study

"Further investigation with larger samples is needed to clarify the Reminder+COM-B message intervention's mechanistic effects on daily adherence in contrast to a daily simple reminder."

"Further investigation is needed to elucidate how AYA perceptions of adherence capability and motivation can be modified via mHealth approaches to facilitate adherence behavior change."

"These hypotheses await further testing in a modified, adaptive version of our mHealth messaging intervention."

"Future investigations that adhere to the Multiphase Optimization Strategy (MOST) framework [30] to systemically and iteratively conduct optimization trials using novel experimental designs (e.g., micro-randomized trials [31]) may help identify specific intervention components delivered at particular times and intensities that maximize intervention efficacy for an individual."

"..future investigators should obtain AYA perspectives on receiving daily reminders only as in the Reminder-only message active control group."

"Future research using similarly rigorous adherence outcome measures is needed to test refined versions of this intervention that incorporate AYA feedback and use study designs aimed at determining the most efficacious intervention components (e.g., micro-randomized trials [31]) to maximize the positive impact on AYAs' medication adherence."

20) Trial limitations, addressing sources of potential bias, imprecision, and, if relevant, multiplicity of analyses

20-i) Typical limitations in ehealth trials

Typical limitations in ehealth trials: Participants in ehealth trials are rarely blinded. Ehealth trials often look at a multiplicity of outcomes, increasing risk for a Type I error. Discuss biases due to non-use of the intervention/usability issues, biases through informed consent procedures, unexpected events.

	1	2	3	4	5	
subitem not at all important	○	○	○	○	◉	essential

Does your paper address subitem 20-i? *

Copy and paste relevant sections from the manuscript (include quotes in quotation marks "like this" to indicate direct quotes from your manuscript), or elaborate on this item by providing additional information not in the ms, or briefly explain why the item is not applicable/relevant for your study

See "Limitations" section header in Discussion.

21) Generalisability (external validity, applicability) of the trial findings

NPT: External validity of the trial findings according to the intervention, comparators, patients, and care providers or centers involved in the trial

21-i) Generalizability to other populations

Generalizability to other populations: In particular, discuss generalizability to a general Internet population, outside of a RCT setting, and general patient population, including applicability of the study results for other organizations

	1	2	3	4	5	
subitem not at all important	○	○	○	◉	○	essential

Does your paper address subitem 21-i?

Copy and paste relevant sections from the manuscript (include quotes in quotation marks "like this" to indicate direct quotes from your manuscript), or elaborate on this item by providing additional information not in the ms, or briefly explain why the item is not applicable/relevant for your study

Our study is a pilot investigation and further study in a full-scale RCT is needed to inform generalizability. We discuss limits of generalizability of the current results in the "Limitations" section.

21-ii) Discuss if there were elements in the RCT that would be different in a routine application setting

Discuss if there were elements in the RCT that would be different in a routine application setting (e.g., prompts/reminders, more human involvement, training sessions or other co-interventions) and what impact the omission of these elements could have on use, adoption, or outcomes if the intervention is applied outside of a RCT setting.

	1	2	3	4	5	
subitem not at all important	○	○	●	○	○	essential

Does your paper address subitem 21-ii?

Copy and paste relevant sections from the manuscript (include quotes in quotation marks "like this" to indicate direct quotes from your manuscript), or elaborate on this item by providing additional information not in the ms, or briefly explain why the item is not applicable/relevant for your study

Our study involved a text messaging intervention for which is delivered remotely and therefore, is not expected to change significantly in how it is implemented outside of a RCT setting.

OTHER INFORMATION

23) Registration number and name of trial registry

Does your paper address CONSORT subitem 23? *

Copy and paste relevant sections from the manuscript (include quotes in quotation marks "like this" to indicate direct quotes from your manuscript), or elaborate on this item by providing additional information not in the ms, or briefly explain why the item is not applicable/relevant for your study

"Trial Registration: ClinicalTrials.gov NCT03651596"

24) Where the full trial protocol can be accessed, if available

Does your paper address CONSORT subitem 24? *

Cite a Multimedia Appendix, other reference, or copy and paste relevant sections from the manuscript (include quotes in quotation marks "like this" to indicate direct quotes from your manuscript), or elaborate on this item by providing additional information not in the ms, or briefly explain why the item is not applicable/relevant for your study

Trial Registration: ClinicalTrials.gov NCT03651596 provides publicly available information about the trial. The research team will provide specific protocol details upon request.

25) Sources of funding and other support (such as supply of drugs), role of funders

Does your paper address CONSORT subitem 25? *

Copy and paste relevant sections from the manuscript (include quotes in quotation marks "like this" to indicate direct quotes from your manuscript), or elaborate on this item by providing additional information not in the ms, or briefly explain why the item is not applicable/relevant for your study

"Acknowledgements: This study was supported by the National Institute of Diabetes and Digestive and Kidney Diseases, National Institutes of Health Ruth L. Kirschstein Postdoctoral Individual National Research Service Award (F32DK118988) and the Society of Pediatric Psychology/American Psychological Association Division 54 Drotar-Crawford Postdoctoral Fellowship Research Grant (both awarded to Cyd K. Eaton, PhD)."

X27) Conflicts of Interest (not a CONSORT item)

X27-i) State the relation of the study team towards the system being evaluated

In addition to the usual declaration of interests (financial or otherwise), also state the relation of the study team towards the system being evaluated, i.e., state if the authors/evaluators are distinct from or identical with the developers/sponsors of the intervention.

	1	2	3	4	5	
subitem not at all important	○	○	○	○	◉	essential

Does your paper address subitem X27-i?

Copy and paste relevant sections from the manuscript (include quotes in quotation marks "like this" to indicate direct quotes from your manuscript), or elaborate on this item by providing additional information not in the ms, or briefly explain why the item is not applicable/relevant for your study

"Conflicts of Interest: None declared."

About the CONSORT EHEALTH checklist

As a result of using this checklist, did you make changes in your manuscript? *

- ○ yes, major changes
- ○ yes, minor changes
- ◉ no

What were the most important changes you made as a result of using this checklist?

N/A

How much time did you spend on going through the checklist INCLUDING making changes in your manuscript *

1 hour

As a result of using this checklist, do you think your manuscript has improved? *

- ○ yes
- ○ no
- ◉ Other: I followed this checklist when writing the paper and therefore, the major points

Would you like to become involved in the CONSORT EHEALTH group?

This would involve for example becoming involved in participating in a workshop and writing an "Explanation and Elaboration" document

- ○ yes
- ◉ no
- ○ Other:

Any other comments or questions on CONSORT EHEALTH

N/A

STOP - Save this form as PDF before you click submit

To generate a record that you filled in this form, we recommend to generate a PDF of this page (on a Mac, simply select "print" and then select "print as PDF") before you submit it.

When you submit your (revised) paper to JMIR, please upload the PDF as supplementary file.

Don't worry if some text in the textboxes is cut off, as we still have the complete information in our database. Thank you!

Final step: Click submit !

Click submit so we have your answers in our database!

Submit

Never submit passwords through Google Forms.

This content is neither created nor endorsed by Google. Report Abuse - Terms of Service - Privacy Policy

Google Forms
